# Supplementary material for: Untwisted restacking of two-dimensional metal-organic framework nanosheets for highly selective isomer separations
Source: Nat Commun. 2019 Jul 2;10:2911. doi: 10.1038/s41467-019-10971-x (PMC6606621; doi:10.1038/s41467-019-10971-x)
Supplement: Supplementary file 1 — Supplementary Info [file 41467_2019_10971_MOESM1_ESM.pdf]

Supplementary Information

Untwisted restacking of two-dimensional metal–organic framework nanosheets for highly selective isomer separations

Tao et al.

Supplementary Methods

Materials and instruments. All of the chemical reagents and solvents were at least of analytical grade and used without further treatment. Zirconium chloride ($ZrCl_4$) and 1, 3, 5-(4-carboxylphenyl)-benzene (H_3BTB) were purchased from J&K Scientific Ltd. (Beijing, China) and Henghua Sci. & Tec. Co., Ltd. (Jinan, China), respectively. Formic acid, alkanes, 1,3,5-trimethylbenzene, n-propylbenzene, isopropybenzene, benzene, toluene, ethylbenzene, n-butylbenzene, ethylbenzene, xylene isomers, chlorotoluene isomers, ethyltoluene isomers, dichlorobenzene isomers, and dibromobenzene isomers were bought from Aladdin Co. Ltd (Shanghai, China). N,N-dimethylformmide (DMF) and ethanol were purchased from Sinopharm Chemical Reagent Co. Ltd (Shanghai, China). Ultrapure water (18 MΩ·cm) from an ELGA purification system (Veolia Water Solutions & Technologies, UK) was used throughout this work.

X-ray diffraction (XRD) patterns were obtained from a Rigaku SmartLab 9 Kw (Tokyo, Japan) diffractometer with a CuKα radiation (1.54056 Å). Data was recorded with 2θ from 4° to 50°. The scanning electron microscope (SEM) images were collected on a JSM-7600 scanning electron microscope (JEOL Ltd.). The high angel annular dark field (HAADF) images of the materials was recorded on an ARM-200CF TEM (JEOL, Tokyo, Japan) operated at 200 keV and equipped with double spherical aberration (Cs) correctors. The attainable resolution of the probe defined by the objective pre-field is 78 picometers. Atomic force microscopy (AFM) measurements were performed with a PicoPlus in tapping mode (Agilent, US). Thermogravimetric analysis (TGA) was collected on a Perkin-Elmer Pyris Diamond 1 TGA analyzer. The diffuse reflectance infrared fourier transform spectroscopy (DRIFTS) of the nanosheets was conducted on a Thermo Fisher Nicolet is 50 ATR-FTIR spectrometer. N_2 sorption measurements were conducted on ASAP 2020 instrument. Proton nuclear magnetic resonance (^{1}HNMR) was recorded on a Bruker AN-400 MHz instrument. All of the separations were performed on an Agilent 7890B gas chromatographic system with a flame ionization detector (FID). Data acquisition and processing were controlled by ChemStation software. Nitrogen (99.999%, Air Liquide, France) was employed as the carrier gas. The inlet temperature of the GC was set to 250 °C, while the temperature of FID was set to 300 °C. A 3 μL analyte was introduced to a 20 mL gastight sealed glass vial and homogenized at 120 °C before the injection for gas chromatographic separation.

Synthesis of 2-D Zr-BTB-BA nanosheets. 2-D Zr-BTB nanosheets with benzoic acid as modulator (Zr-BTB-BA nanosheets) were synthesized as follows. A mixture of $ZrCl_4$ (10 mg) and H_3BTB (10 mg) were dissolved in 3 mL DMF in a 22 mL pyrex vial under ultrasonication for 10 min. Benzoic Acid (BA, 600 mg) and water (250 μL) were then added to the clear solution. The vial was sealed and placed in an oven at 120 °C for 24 h, then cooled to room temperature. The white solids were obtained by centrifugation, then washed with DMF and ethanol. To avoid the restacking between nanosheets, the as-synthesized products were kept in ethanol solution, and marked as twisted Zr-BTB-BA nanosheets.

Synthesis of 2-D Zr-BTB-PABA nanosheets. 2-D Zr-BTB-FA nanosheets were post-synthetically modified with 4-aminobenzoic acid to obtain 2-D Zr-BTB-PABA nanosheets. The 2-D Zr-BTB-FA nanosheets (around 100 mg) was stirred in 30 mL DMF solution, to which a solution of 4-aminobenzoic acid (PABA, 1000 mg) in 20 mL DMF was introduced. This mixture was refluxed for 72 h. After cooling down to room temperature, the yellowish products were collected by centrifugation, washed with DMF and ethanol. To avoid the restacking between nanosheets, the as-synthesized products were kept in ethanol solution, and marked as twisted Zr-BTB-PABA nanosheets.

Synthesis of 2-D restacked untwisted Zr-BTB-BA and Zr-BTB-PABA nanosheets. The as-synthesized 2-D Zr-BTB-BA and Zr-BTB-PABA nanosheets were heated under vacuum at 80 °C for 6 h to obtain 2-D Zr-BTB-BA and Zr-BTB-PABA nanosheets with restacked untwisted structures. To distinguish with twisted Zr-BTB-FA nanosheets, restacked sample was marked as untwisted Zr-BTB-BA nanosheets and untwisted Zr-BTB-PABA nanosheets.

Synthesis of Zr-BTB-FA micrometer-sized nanoplates. The synthesis condition for Zr-BTB-FA nanosheets was changed by increasing amount of formic acid, resulting in the morphology changes from nanosheets to micrometer-sized plates. In brief, H_3BTB (12.5 mg) and $ZrCl_4$ (10.12 mg) were dissolved in 5 mL DMF in a 22 mL Pyrex vial under ultrasonication for 10 min. Formic aid (1.910 g) and water (60 μL) were then added to the clear solution, and kept at 120 °C for 48 h. White Zr-BTB-FA micrometer-sized plates were obtained and then dried under a dynamic vacuum at 80 °C for 12 h. To compare the separation performance, the Zr-BTB-FA micrometer-sized plates coated capillary column was obtained by the same method described above.

Synthesis of 3-D Zr-BTB. The 3-D Zr-BTB was also synthesized according to a literature procedure[1]. Typically, H_3BTB (110 mg, 0.25 mmol) and $ZrCl_4$ (120 mg, 0.50 mmol) were dissolved in mixed solvent DMF and acetic acid (v/v =1 : 3, 16 mL). Then it was slowly heated to 120 °C from room temperature in 8 hours, kept at 120 °C for 72 hours, and then slowly cooled to 30 °C in 10 hours. The white crystals were harvested by washing with DMF and ethanol, sequentially. The product was dried at 80 °C in a vacuum oven for 6 hours. To compare the separation performance, the 3-D Zr-BTB MOFs coated capillary column was obtained by the same method described above.

PXRD Simulation. The construction of the periodic twisted structures with specific angles (8°, 14° and 30°) of 2-D Zr-BTB-FA nanosheet stackings was not successful due to the failure of adding periodic boundary conditions to the simulated supercell. Instead, we compared the PXRD patterns for twisted and untwisted experimental results with the simulated untwisted structure (Fig. 3a and b).

Preparation of Zr-BTB coated capillary columns. A fused silica capillary (30 m long × 0.25 mm i.d., Yongnian Optic Fiber Plant, Hebei, China) was pre-treated according the following recipe before dynamic coating with the nanosheets: the capillary was washed sequentially with 1 $mol \cdot L^{-1}$ NaOH for 2 h, ultrapure water for 30 min, 0.1 $mol \cdot L^{-1}$ HCl for 2 h, ultrapure water again until the outflow reached pH=7.0, and finally methanol for 30 min. After the above process, the capillary was modified with 3-aminopropyltriethoxysilane (APTES) to provide the amino groups to enhance the interactions with nanosheets on the inner wall of capillary column. The pretreated capillary was filled with a methanolic solution of APTES (50%, v/v), and incubated in a 40 °C water bath overnight with both ends of the capillary sealed. The APTES-modified capillary was rinsed with methanol to flush out the residuals, and dried with a stream of nitrogen at 120 °C.

2-D twisted Zr-BTB-FA nanosheets were coated onto the pretreated capillary column by a simple dynamic coating method as follows: 1 mL (3.33 $mg \cdot mL^{-1}$) ethanol suspension of 2-D twisted Zr-BTB-FA was first filled into the capillary column and then pushed through the column at a velocity of 30 $cm \cdot min^{-1}$ to leave a wet coating layer on the inner wall of the capillary column. To avoid acceleration of the solution plug near the end of the column, 1-m long buffer tube was attached to the capillary column end as a restrictor. After coating, the capillary column was settled for conditioning under nitrogen for 2 h to remove the solvent. Further conditioning of the capillary column

was carried out using a temperature program: 30 °C for 30 min, ramp from 30 °C to 250 °C at a rate of 1 °C min^{-1} and 250 °C for 240 min. The temperature program was repeated for 3 times. The twisted Zr-BTB-BA and Zr-BTB-PABA nanosheets coated capillary column were obtained with the same method. Three restacked untwisted Zr-BTB nanosheets samples with different modulators or spacing ligands were also coated onto the capillary columns to obtain the untwisted Zr-BTB-FA, Zr-BTB-BA and Zr-BTB-PABA nanosheets coated capillary columns according to the same method.

Calculation of selectivity and resolution. The selectivity factor ($\alpha_{B/A}$) for analytes A and B on the capillary column were calculated from gas chromatogram according to Supplementary Equation 1:

$$\alpha_{B/A} = \frac{t_B - t_0}{t_A - t_0} \qquad \text{Supplementary Equation 1}$$

Where t_A, t_B and t_0 are the retention time of analytes A, B and unretained compound, respectively, under the same operation conditions.

The resolution (R) for analytes A and B on the capillary column were calculation according to Supplementary Equation 2:

$$R_s = \frac{t_B - t_A}{1/2(w_{B+}w_A)} \qquad \text{Supplementary Equation 2}$$

Where w_A and w_B are the peak width of analytes A and B, respectively.

Calculation of thermodynamic parameters. The enthalpy change (ΔH) and entropy change (ΔS) for the transfer of analytes from the mobile to the stationary phase were calculated from the van't Hoff equation (3):

$$ln\, k' = -\frac{\Delta H}{RT} + \frac{\Delta S}{R} + ln\phi \qquad \text{Supplementary Equation 3}$$

Where k' is the retention factor, R is the gas constant, T is the absolute temperature, and ϕ is the phase ratio (the ratio of the volume of the stationary phase (Vs) to that of the mobile phase (V_m)). To obtain ϕ, Vs was calculated from the film thickness of nanosheets coated capillary column, while V_m was calculated from the column internal volume subtract the Vs. Thus, the lnϕ of untwisted and twisted columns were determined to be -4.227 and -5.742. The k' was calculated according to Supplementary Equation 4:

$$k' = \frac{t - t_0}{t_0} \qquad \text{Supplementary Equation 4}$$

Where t_R is the retention time of the analyte, and t_0 is the retention time of an unretained compound on the column.

Supplementary Figures

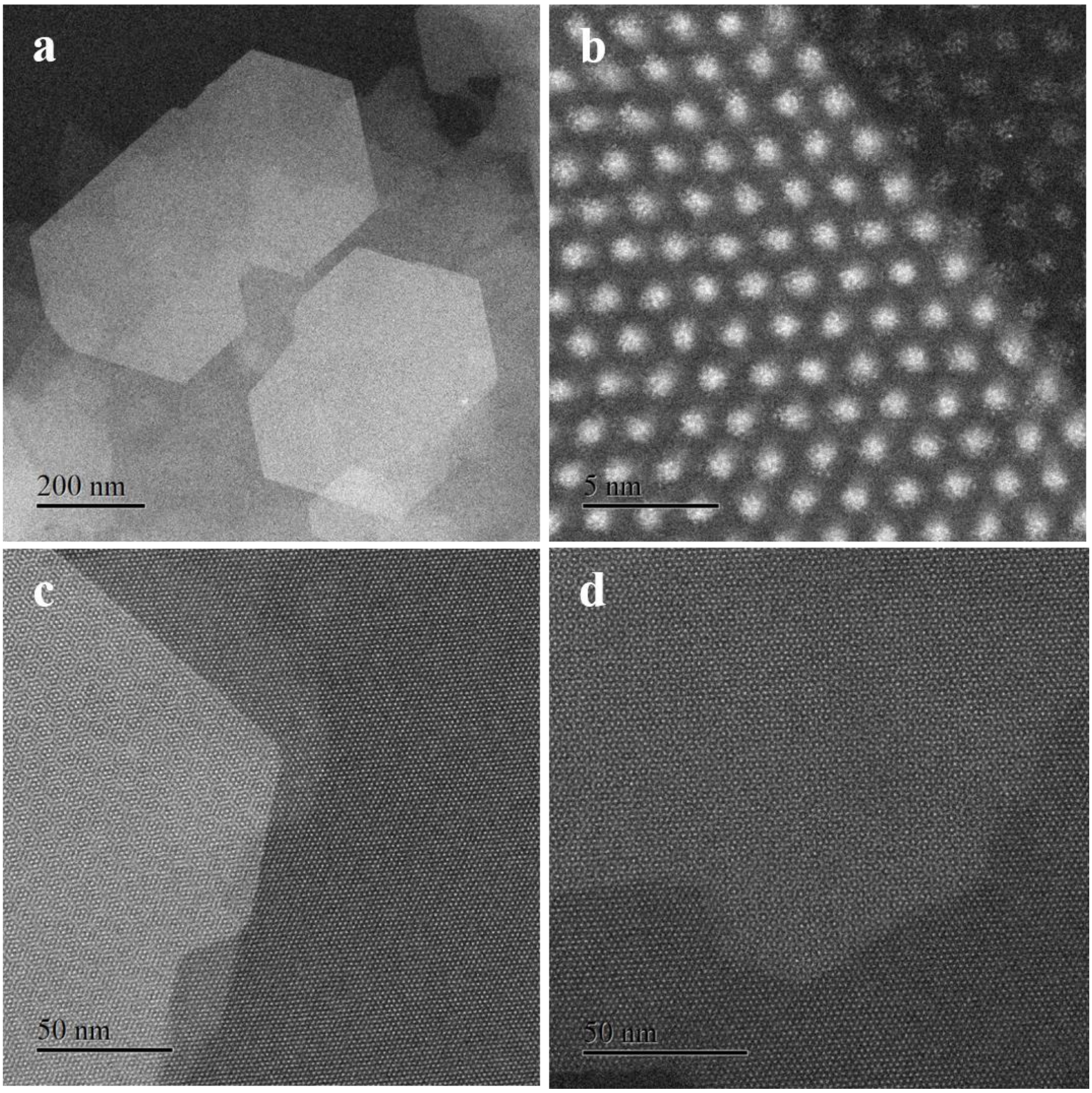

Supplementary Figure 1. (a) and (b) HRTEM images of 2-D twisted Zr-BTB-FA nanosheets with different magnifications, (c) and (d) HAADF images with Moiré patterns, indicating twisted stackings of adjacent nanosheets.

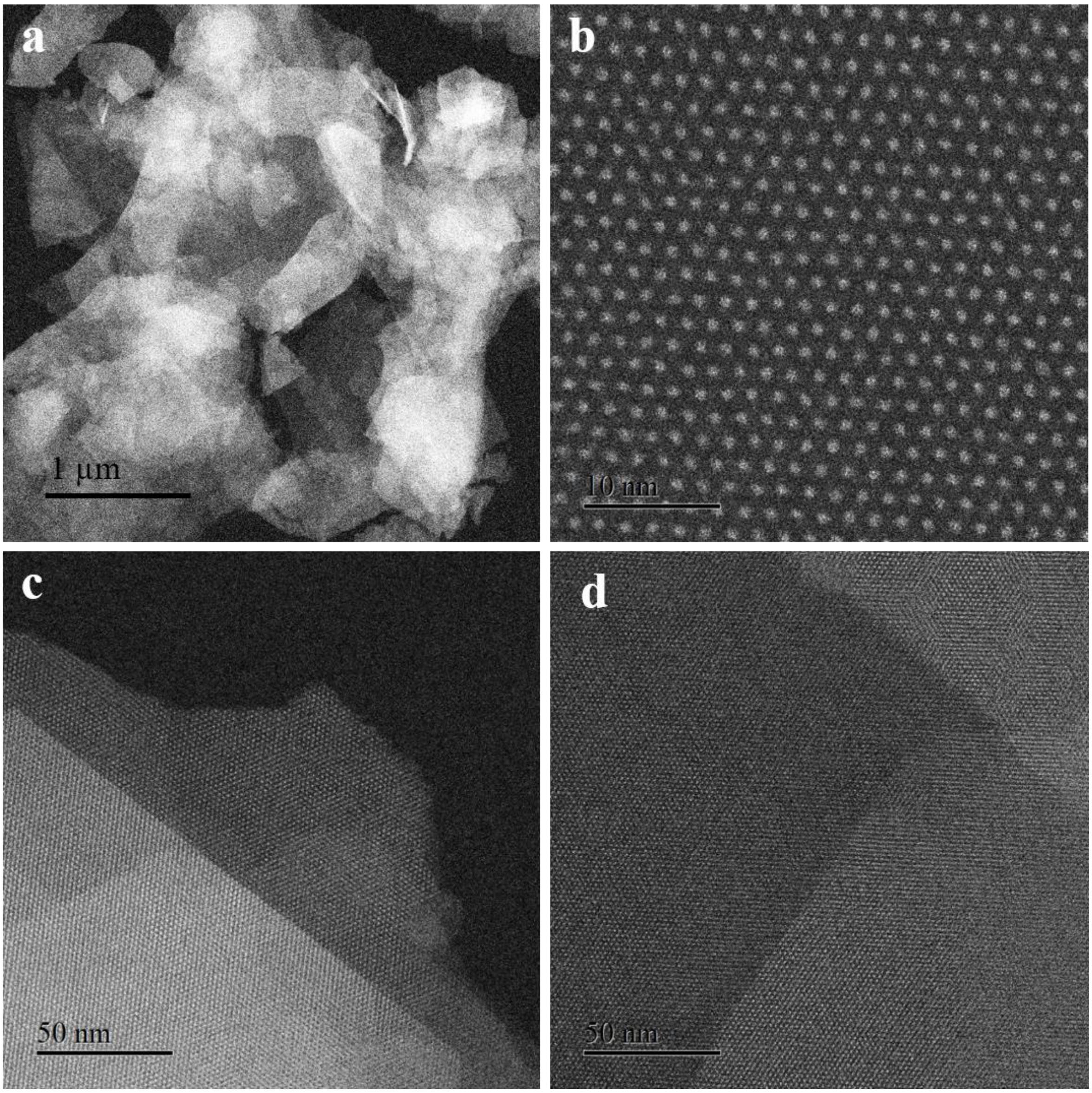

Supplementary Figure 2. (a) and (b) HRTEM images of 2-D untwisted Zr-BTB-FA nanosheets with different magnifications, (c) and (d) HAADF images without Moiré patterns, indicating untwisted stackings of adjacent nanosheets.

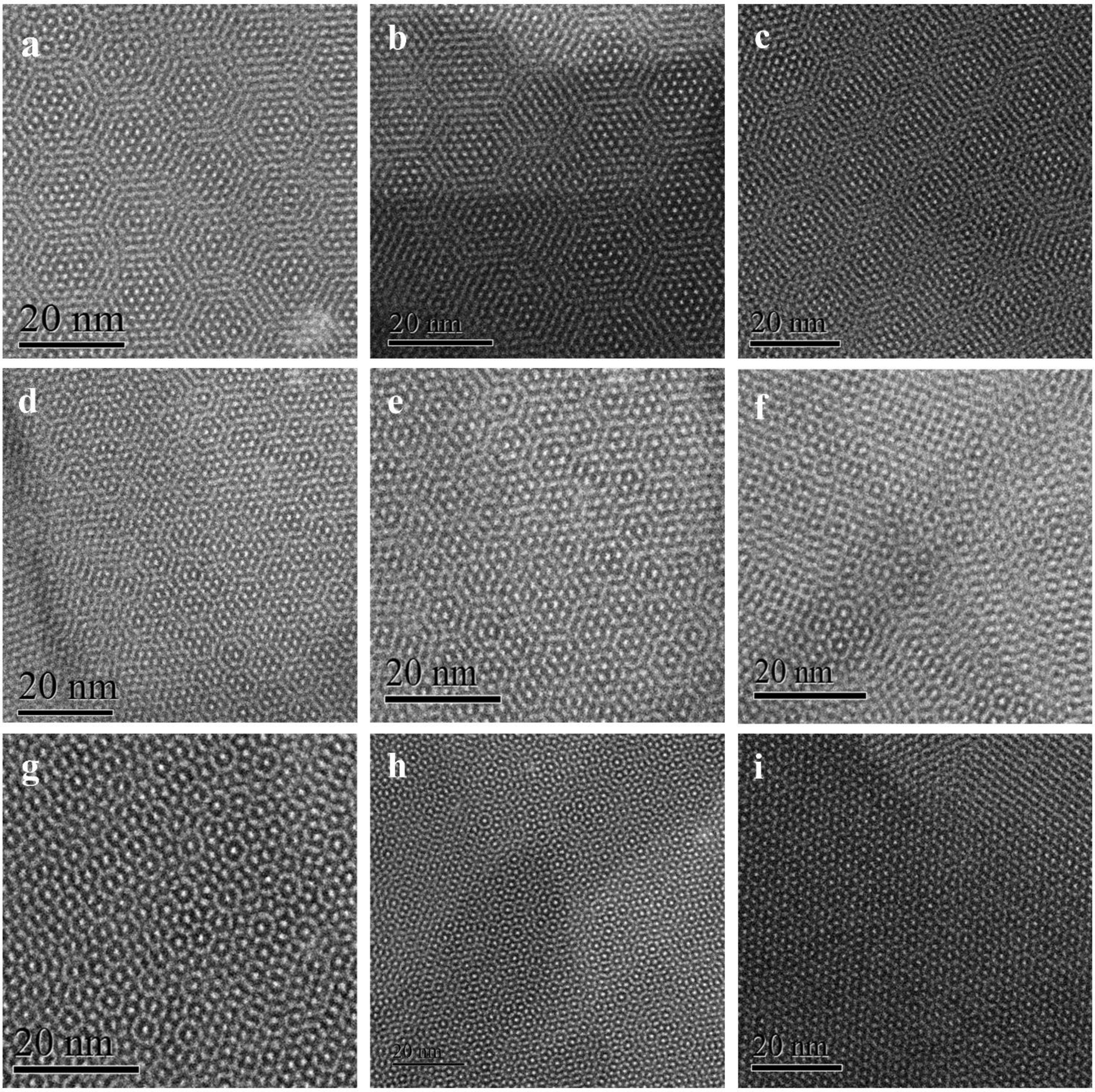

Supplementary Figure 3. HAADF images of 2-D twisted Zr-BTB-FA nanosheets with Moiré patterns. (a, b and c) twisted Zr-BTB-FA nanosheets with the rotation of 8º, (d and e) twisted Zr-BTB-FA nanosheets with the rotation of 14º, (f, g, h and i) twisted Zr-BTB-FA nanosheets with the rotation of 30º. In order to simulate environment in the capillary, the sample was paved on a watch glass and placed in the GC oven to experienced temperature program for 250 ºC before HRTEM measurements.

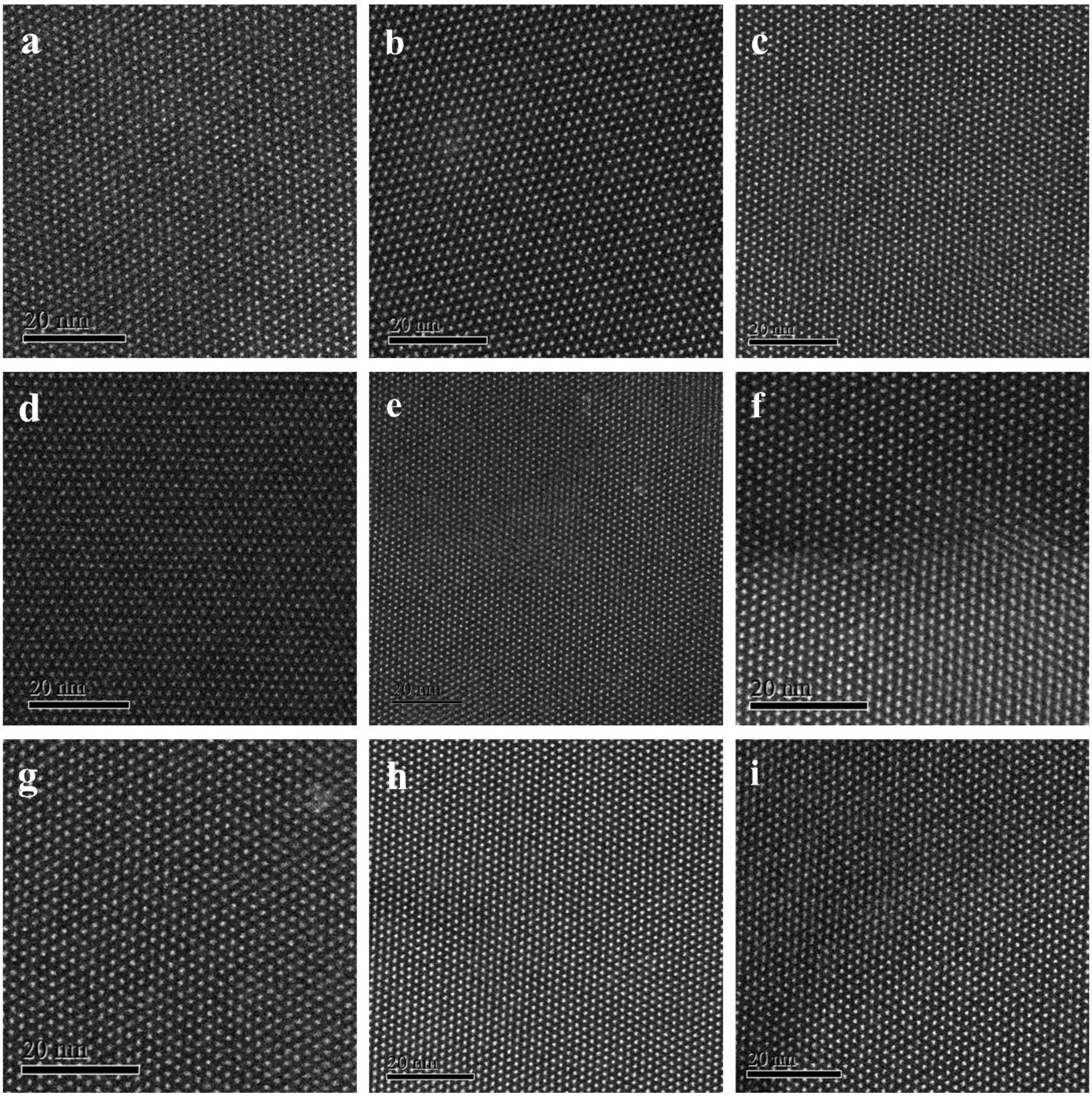

Supplementary Figure 4. (a-i) HAADF images of 2-D untwisted Zr-BTB-FA nanosheets without Moiré patterns. In order to simulate environment in the capillary, the sample was paved on a watch glass and placed in the GC oven to experienced temperature program for 250 ºC before HRTEM measurements.

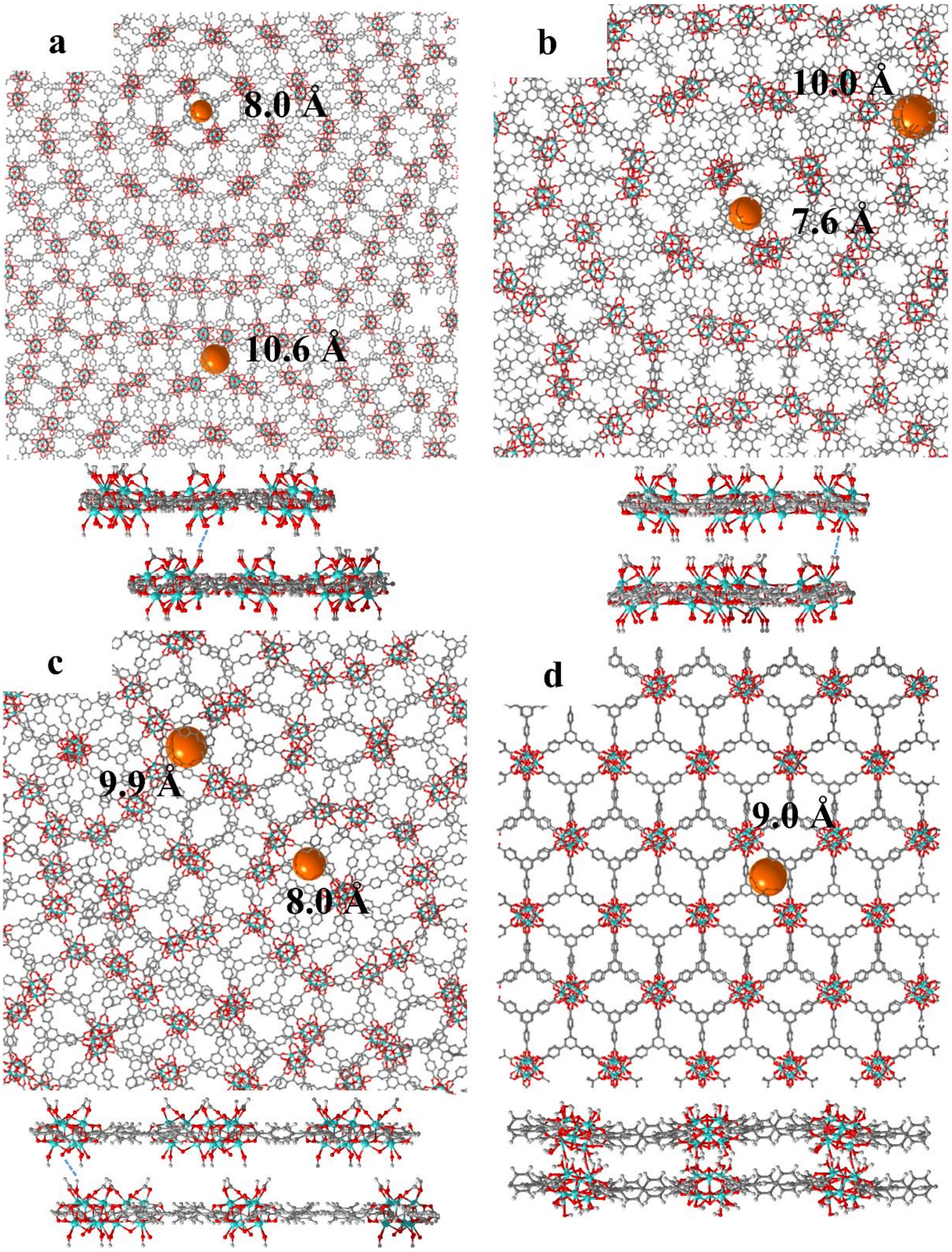

Supplementary Figure 5. The predictable pore size of 2-D simulated Zr-BTB-FA structure and the sideviews between the adjacent layers. (a) twisted Zr-BTB-FA with the rotation of 8º, (b) twisted Zr-BTB-FA with the rotation of 14º, (c) twisted Zr-BTB-FA with the rotation of 30º, (d) untwisted Zr-BTB-FA with the rotation of 0º.

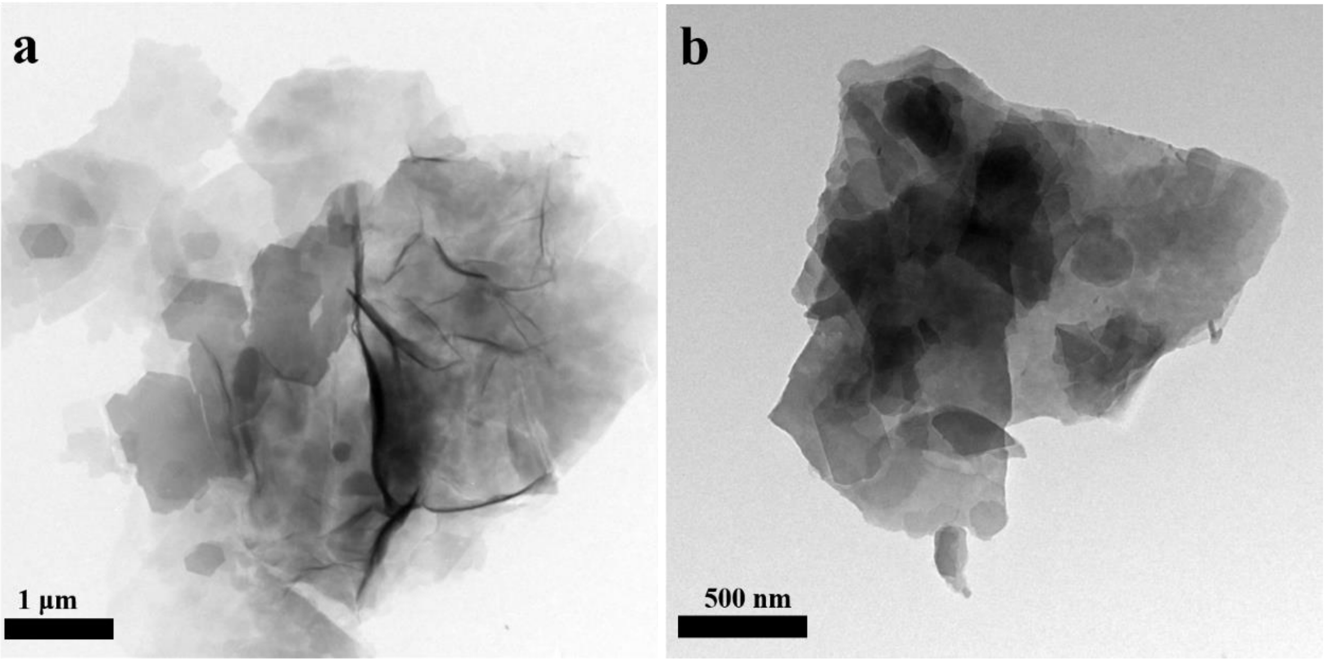

Supplementary Figure 6. TEM images of as-synthesized (a) twisted Zr-BTB-FA nanosheets, (b) untwisted Zr-BTB-BA nanosheets.

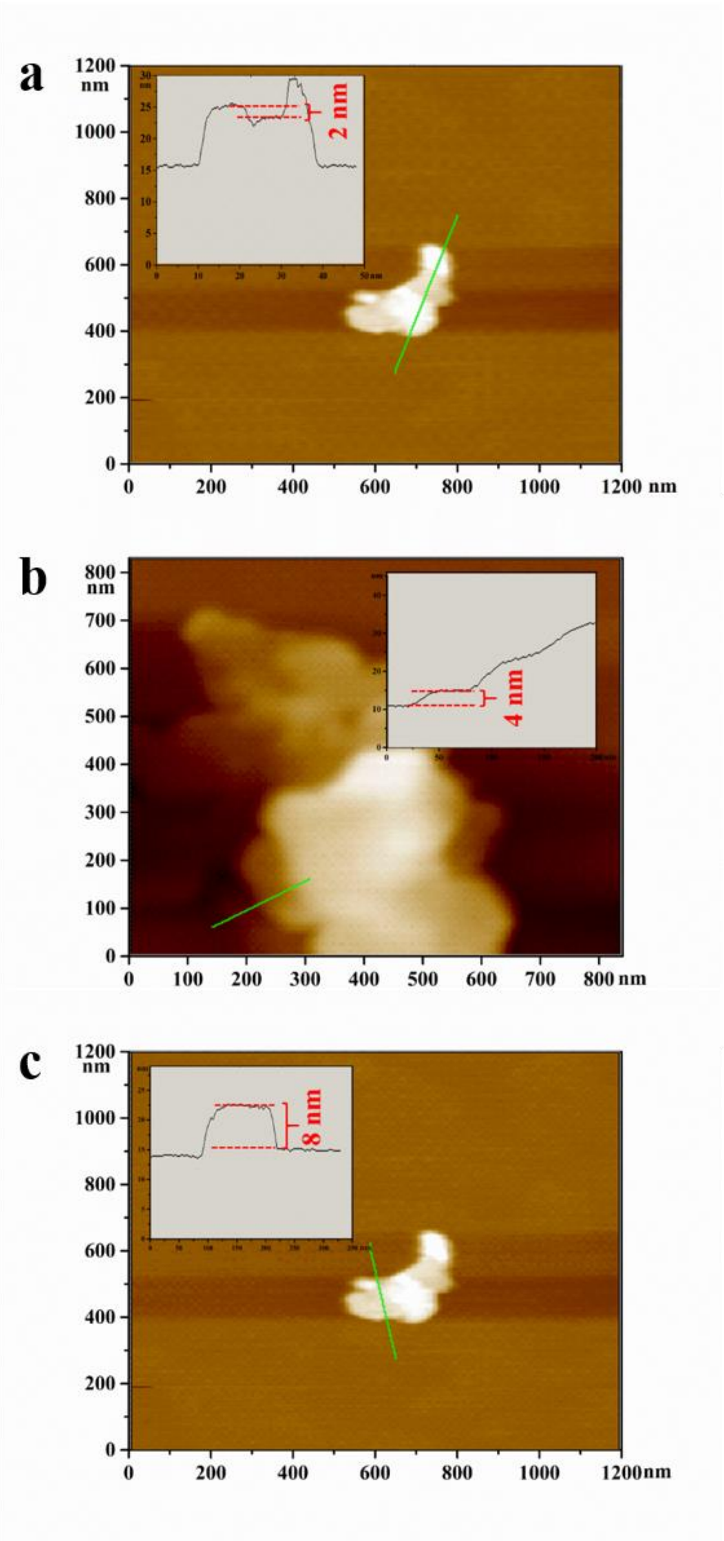

Supplementary Figure 7. AFM images of as-synthesized 2-D twisted Zr-BTB-FA nanosheets, demonstrating the nanosheets stacking with the heights of (a) 2 nm, (b) 4 nm and (c) 8 nm, respectively.

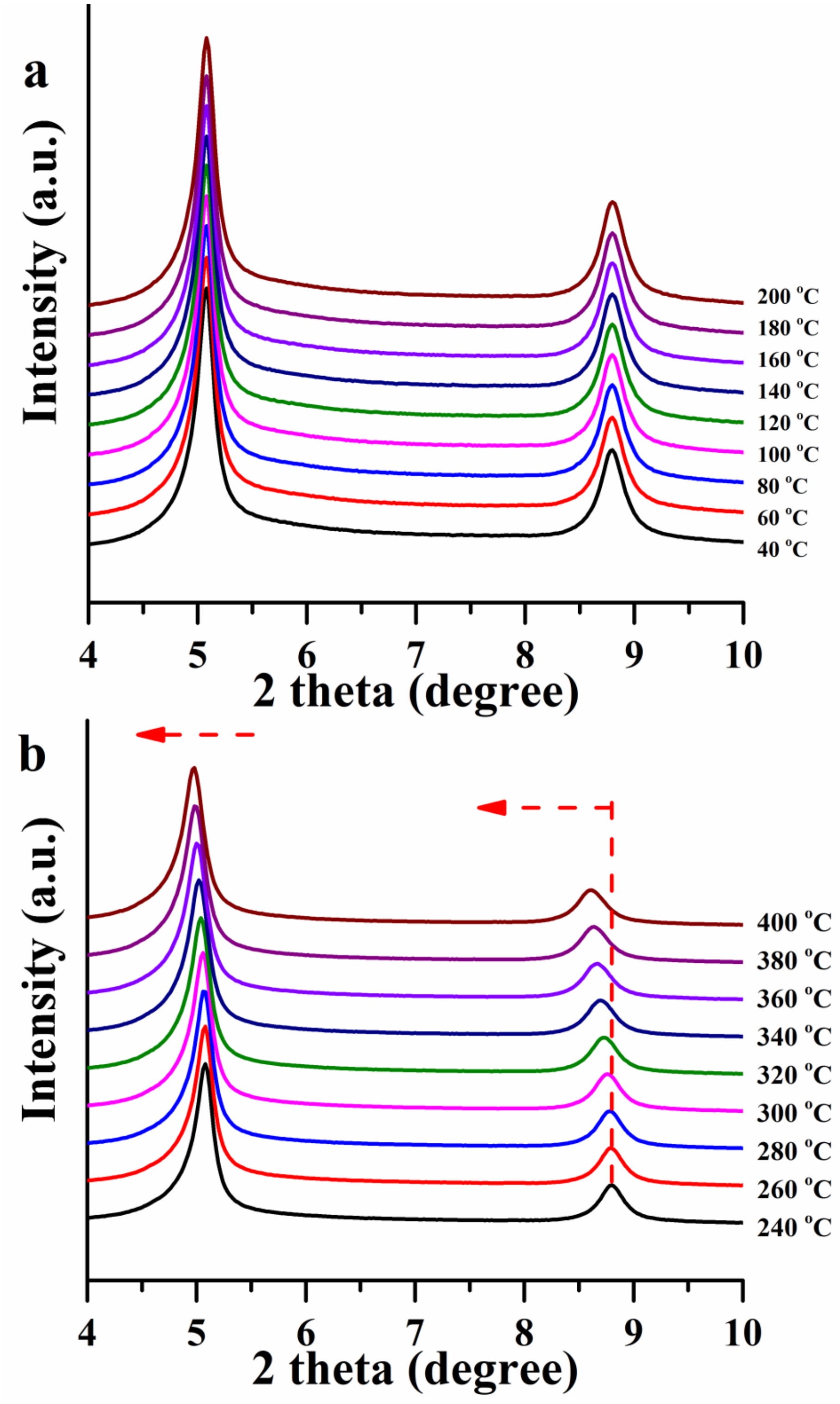

Supplementary Figure 8. The *in-situ* variable temperature PXRD patterns of 2-D Zr-BTB-FA nanosheets. Measurement procedures: Initial heating at 250 ºC for 6 hours, then heating temperature programing from (a) 40 ºC to 200 ºC and from (b) 240 ºC to 400 ºC at the rate of 1 ºC·min^{-1}. The shift of diffraction peaks towards low degree region was observed, while the intensities of diffraction were well kept, indicating highly crystalline MOFs with small lattice expansion under high temperature.

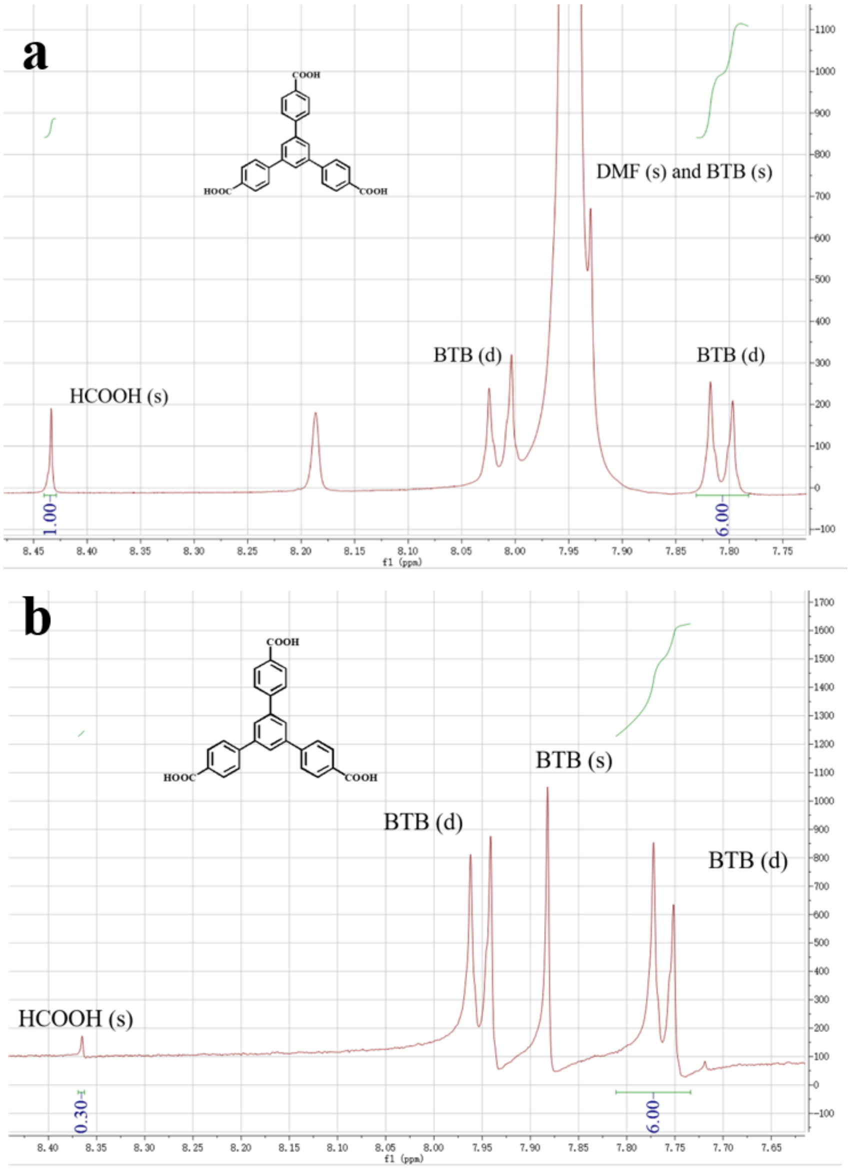

Supplementary Figure 9. H^1NMR of 2-D (a) twisted Zr-BTB-FA and (b) twisted Zr-BTB-FA experienced temperature program 250 ºC. Molar ratios of n(HCOOH): n(BTB)=1: 1 and 0.3: 1 for twisted Zr-BTB-FA and twisted Zr-BTB-FA experienced temperature program 250 ºC were obtained from the peak integration, respectively. Considering the coordination of Zr_6 cluster, the molar ratios of n(Zr_6 cluster): n(HCOOH): n(BTB)=1: 2: 2 and n(Zr_6 cluster): n(HCOOH): n(BTB)=1: 0.6: 2, respectively. This phenomenon indicated the loss of HCOOH from 2 to 0.6 per Zr_6 cluster during the temperature program. The nanosheets samples were digested in d_6-DMSO/ K_3PO_4-D_2O (4:1) before H^1NMR measurement.

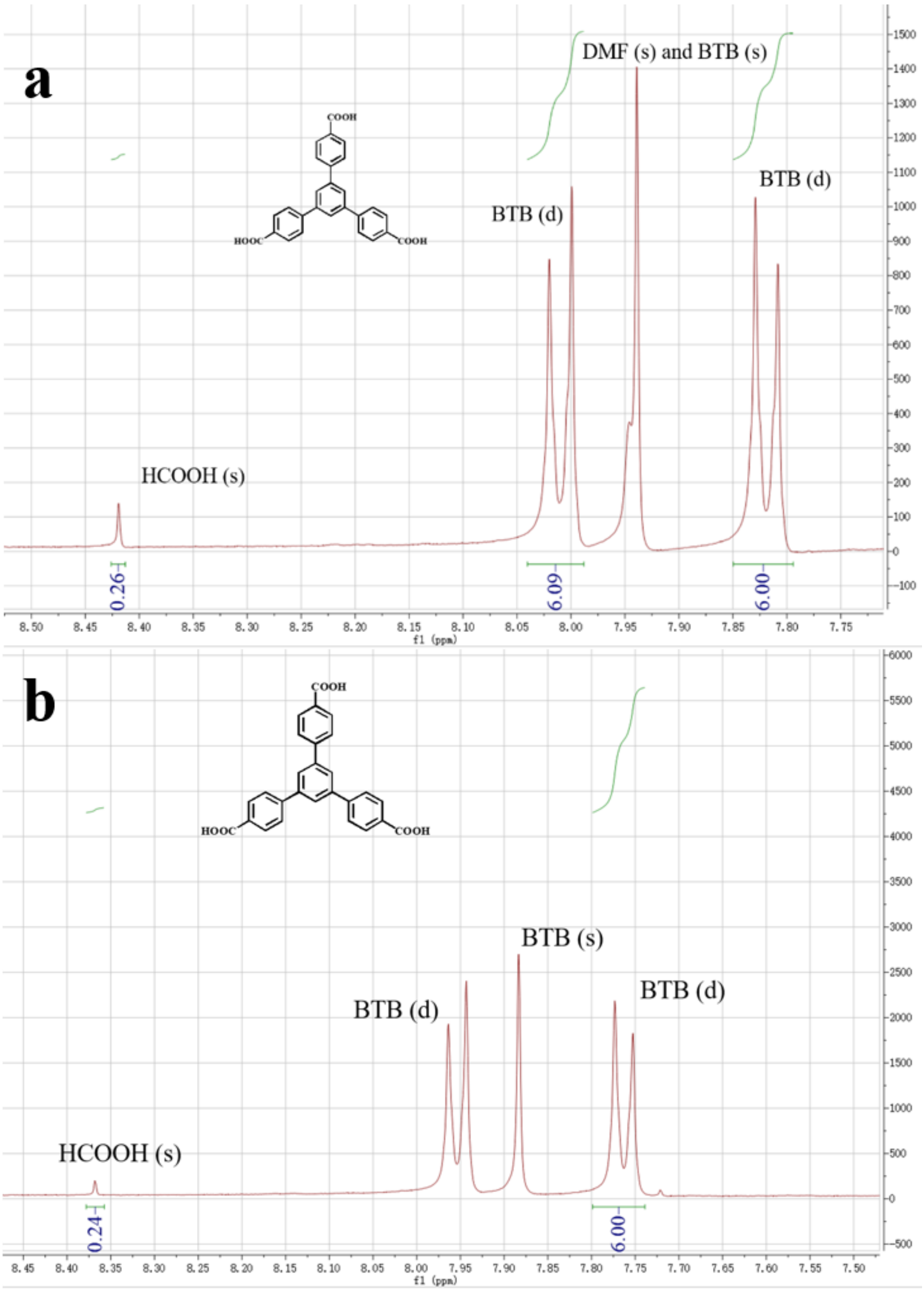

Supplementary Figure 10. H^1NMR of 2-D (a) untwisted Zr-BTB-FA and (b) untwisted Zr-BTB-FA experienced temperature program 250 ºC. Molar ratios of n(HCOOH): n(BTB)=1: 0.26 and 0.3: 0.24 for untwisted Zr-BTB-FA and untwisted Zr-BTB-FA experienced temperature program 250 ºC were obtained from the peak integration, respectively. Considering the coordination of Zr_6 cluster, the molar ratios of n(Zr_6 cluster): n(HCOOH): n(BTB)=1: 0.52: 2 and n(Zr_6 cluster): n(HCOOH): n(BTB)=1: 0.48: 2, respectively. This phenomenon indicated no loss of HCOOH during the temperature program. The nanosheets samples were digested in d_6-DMSO/ K_3PO_4-D_2O (4:1) before H^1NMR measurement.

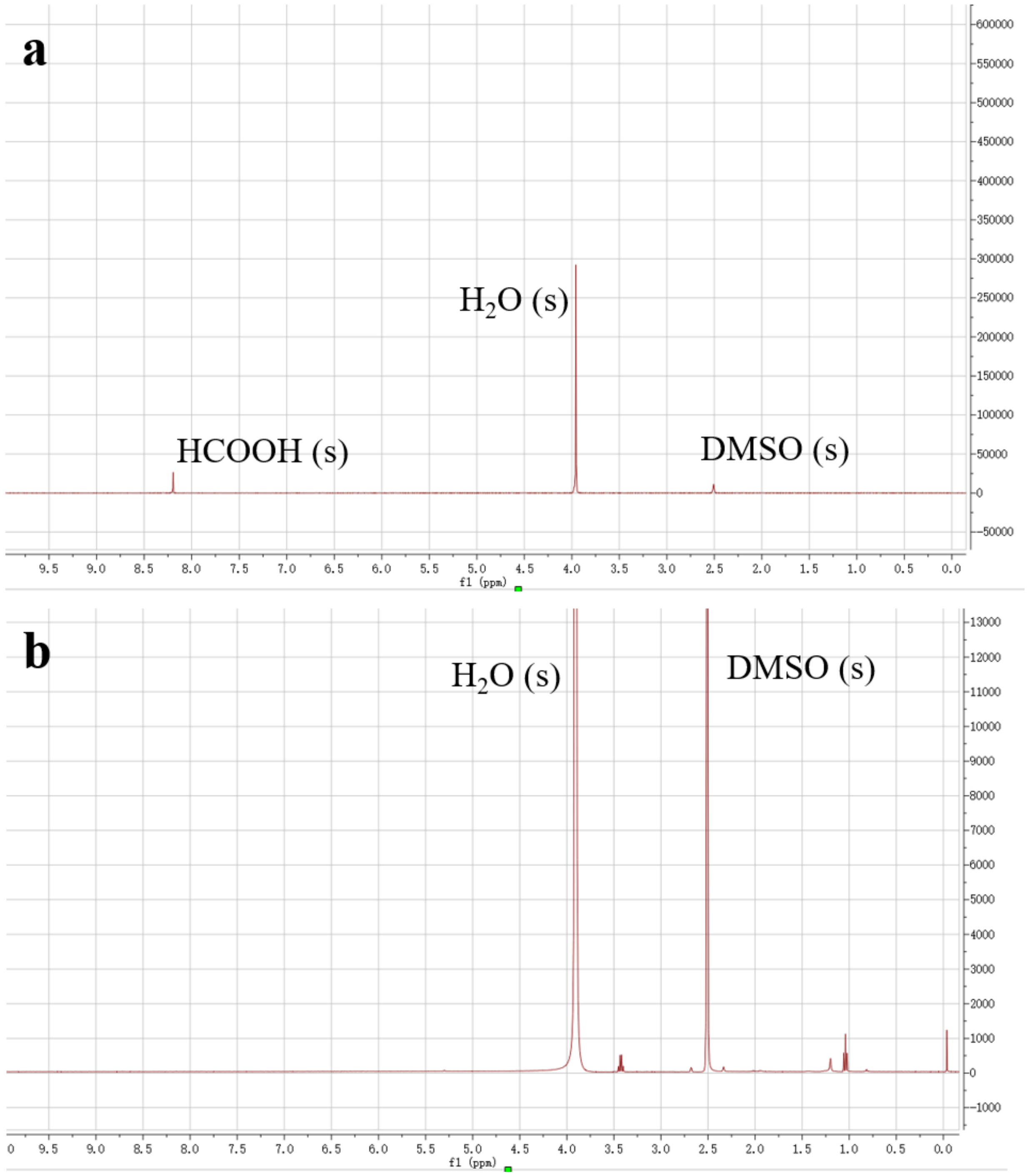

Supplementary Figure 11. H^{1}NMR of 2-D (a) 2.6 mmol·L^{-1} HCOOH and (b) the last ethanol washing eluent for twisted Zr-BTB-FA nanosheets. There is no HCOOH in the washing eluent, indicating no trapped FA in the Zr-BTB nanosheets.

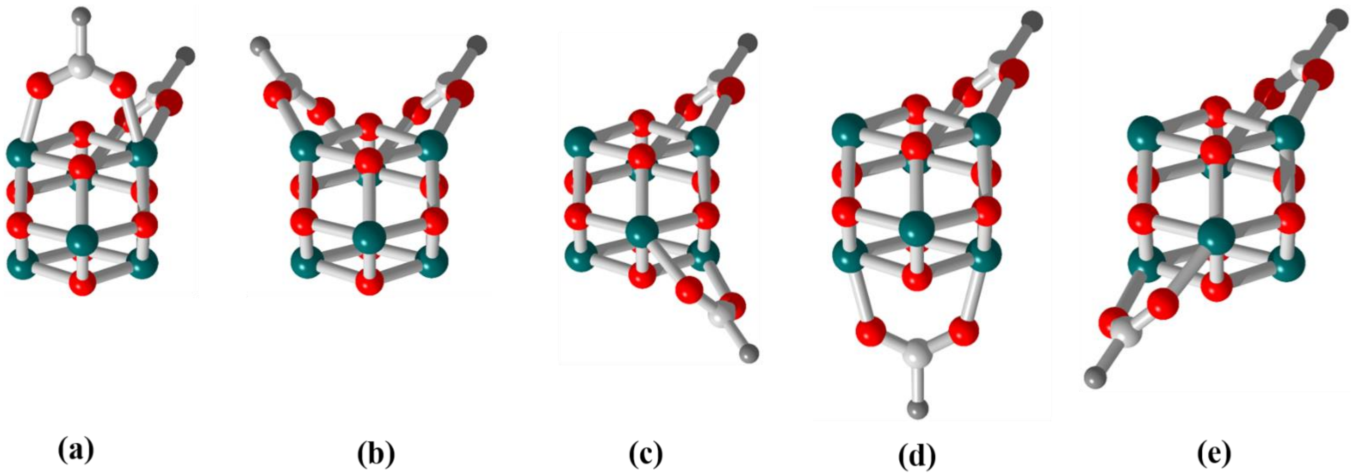

Supplementary Figure 12. The five arrangement possibilities of two HCOOH molecules on the six uncoordinated sites of Zr_6 cluster in Zr-BTB nanosheets. The asymmetrical arrangements of two FA molecules (a-d) in one Zr_6 clusters could possibly provide driving forces to form the twisted stacking with different angles.

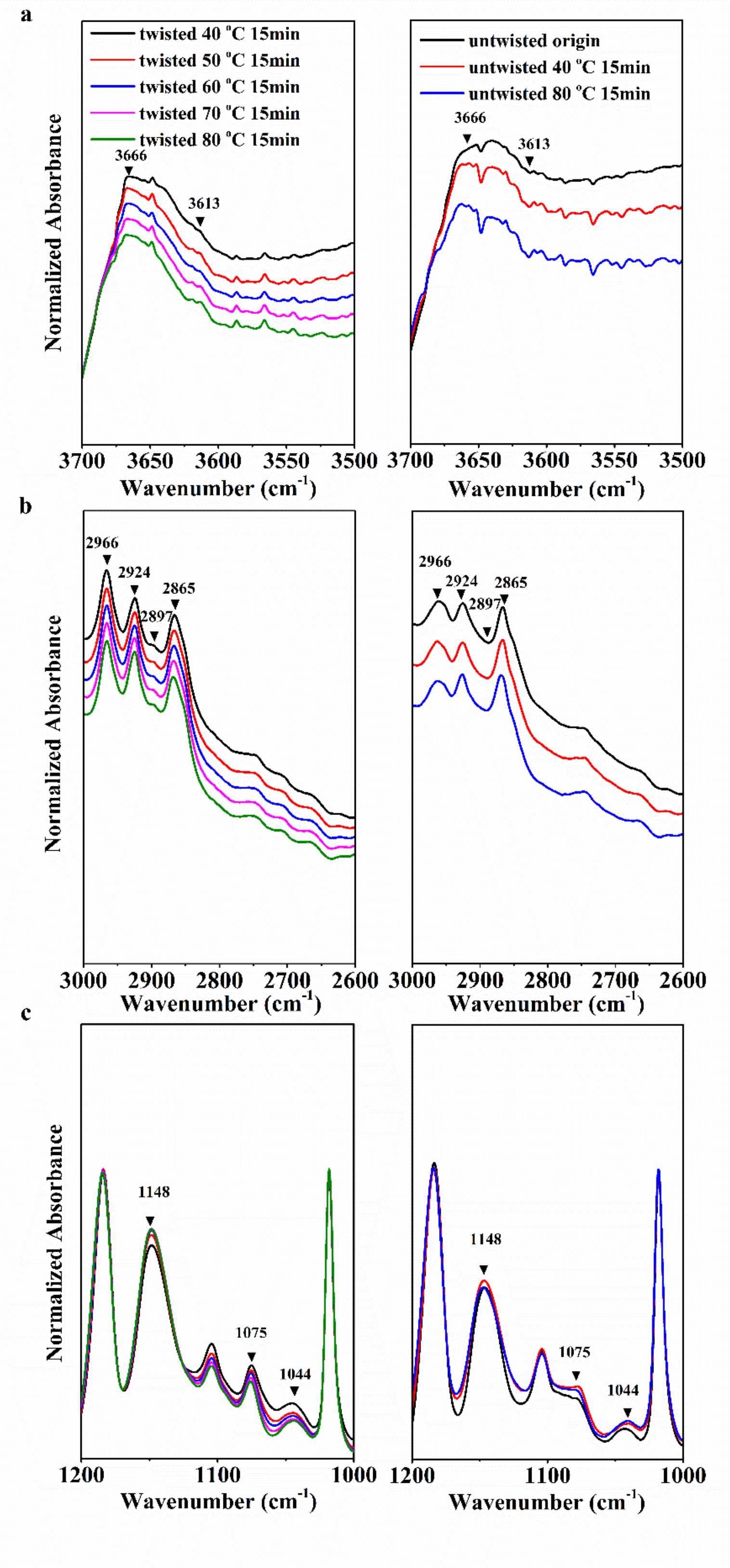

Supplementary Figure 13. DRIFTS spectra of twisted (left) and untwisted (right) Zr-BTB-FA in the range of (a) 3500-3700 cm^{-1}, (b) 2600-3000 cm^{-1} and (c) 1000-1200 cm^{-1}. To confirm the differences between the two stacking modes of Zr-BTB-FA nanosheets, the ex-situ DRIFTS were measured (Supplementary Figure 7). As reported, the

non-hydrogen-bonded μ_3-OH and terminal -OH and OH_2 stretches of Zr_6 cluster had a strong absorption at around 3674 cm^{-1}, while, we observed there was no absorption in this range for both twisted and untwisted Zr-BTB-FA[2]. The phenomenal was reasonable because the terminal -OH and OH_2 could easily be replaced by the ethoxy groups which were introduced from the washing procedure, leading to the red shift of μ_3-OH stretches absorption at 3674 cm^{-1} to lower wavenumber to form a broader band[2]. Obviously, the vibration of the O–H bonds around 3613 cm^{-1} almost disappeared in untwisted Zr-BTB-FA compared with twisted Zr-BTB-FA, indicating that this kind of O-H bonds only existed in twisted Zr-BTB-FA. Meanwhile, as observed, the ethoxy groups was unavoidable in both twisted and untwisted Zr-BTB-FA because of the washing treatment. The C-H bands for ethoxy groups appeared in the spectra at 2966 cm^{-1}, 2924 cm^{-1} and 2865 cm^{-1}, respectively. The relative absorption at 2966 cm^{-1} decreased with the increase of *in-situ* temperature for twisted Zr-BTB-FA. We supposed that the ethoxy groups on the Zr_6 cluster would dissociate through the heating treatment, so there would be less ethoxy groups in untwisted Zr-BTB-FA than in twisted Zr-BTB-FA. This hypothesis could also be supported by the decrease of the absorption of C-O bands at 1148 cm^{-1} in untwisted Zr-BTB-FA. Although it had been few discussed in the previous articles, the absorptions at 1075 cm^{-1} and 1044 cm^{-1} were also from ethoxy groups, which decreased in untwisted Zr-BTB-FA, representing less ethoxy groups. Furthermore, the absorptions at 2897 cm^{-1} represented the C-H stretching vibrations of the formate ligands which were introduced by the adding of the modulators or the decomposition of the DMF molecules[3]. Obviously, in the untwisted Zr-BTB-FA, the vibrations of the C-H were not observed. We supposed that the formate ligands bond to the Zr_6 cluster in twisted Zr-BTB-FA exactly existed, while the formate ligands bond in untwisted Zr-BTB-FA was out of the detection limit of the DRIFTS.

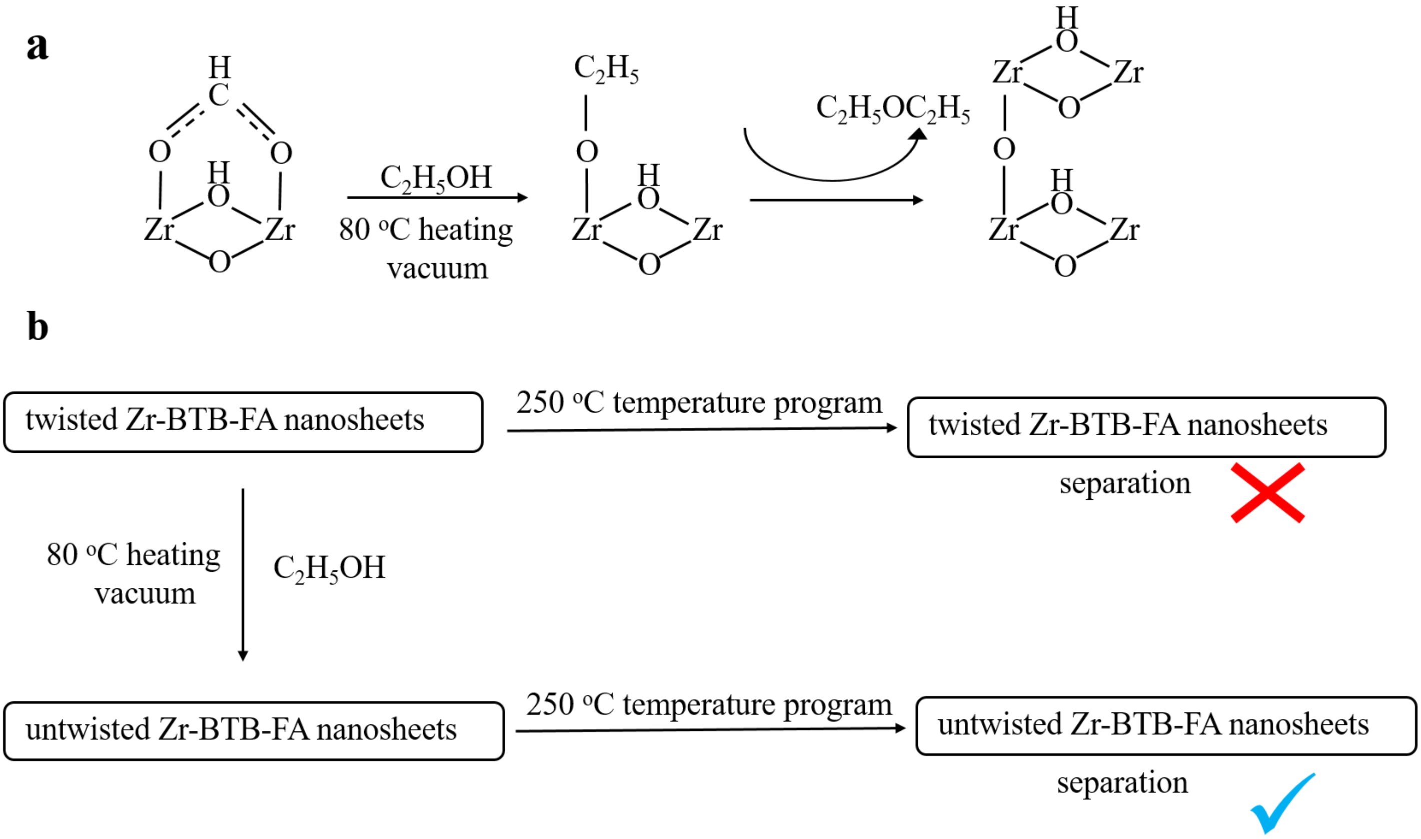

Supplementary Figure 14. (a) possible mechanism of chemical linkages of Zr-O-Zr between Zr-clusters in the adjacent layers through the ethoxy group-mediated linkage reaction, (b) schematic transformation of twisted Zr-BTB-FA to untwisted Zr-BTB-FA. Their respective morphologies and separation abilities were checked after this transformation. The check mark and cross mark indicate good and bad separation results, respectively.

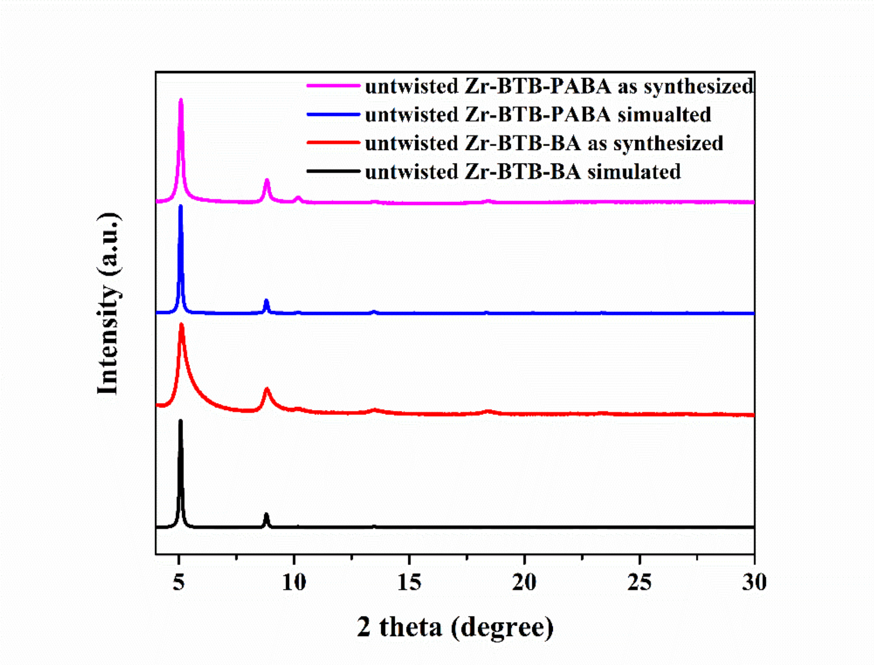

Supplementary Figure 15. PXRD of as-synthesized 2-D untwisted Zr-BTB-BA and Zr-BTB-PABA nanosheets.

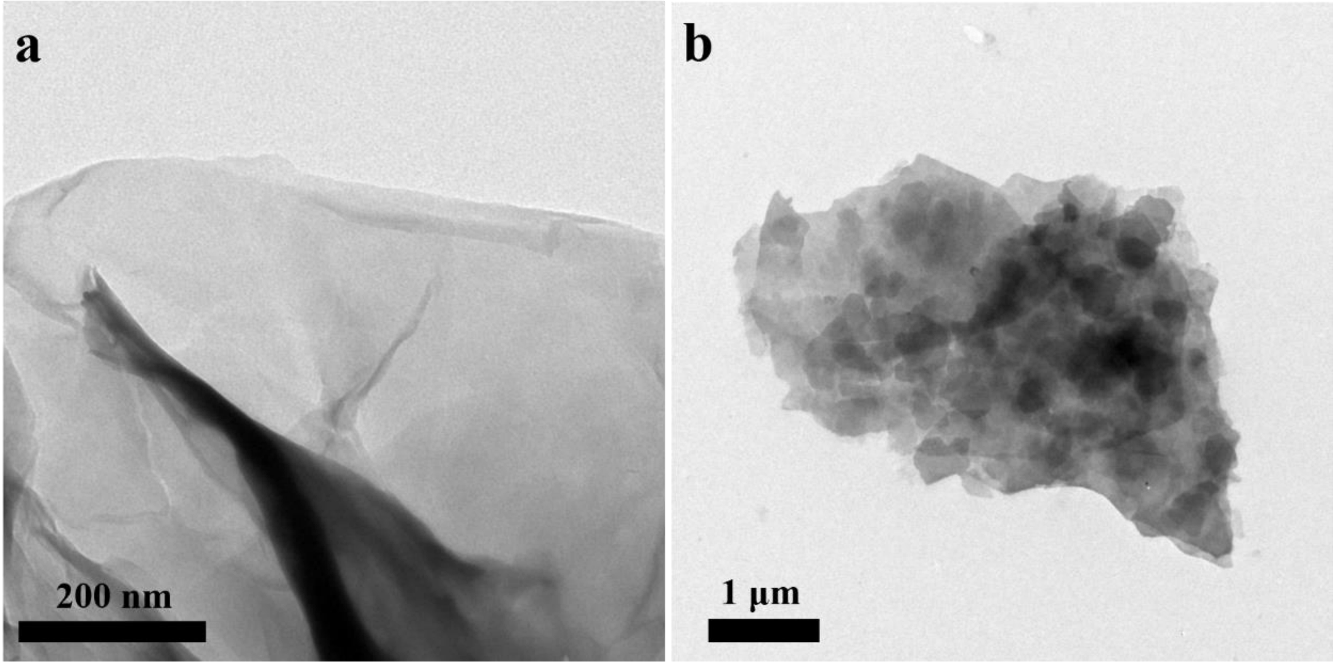

Supplementary Figure 16. TEM images of (a) as-synthesized Zr-BTB-BA nanosheets and (b) untwisted Zr-BTB-BA nanosheets after heating.

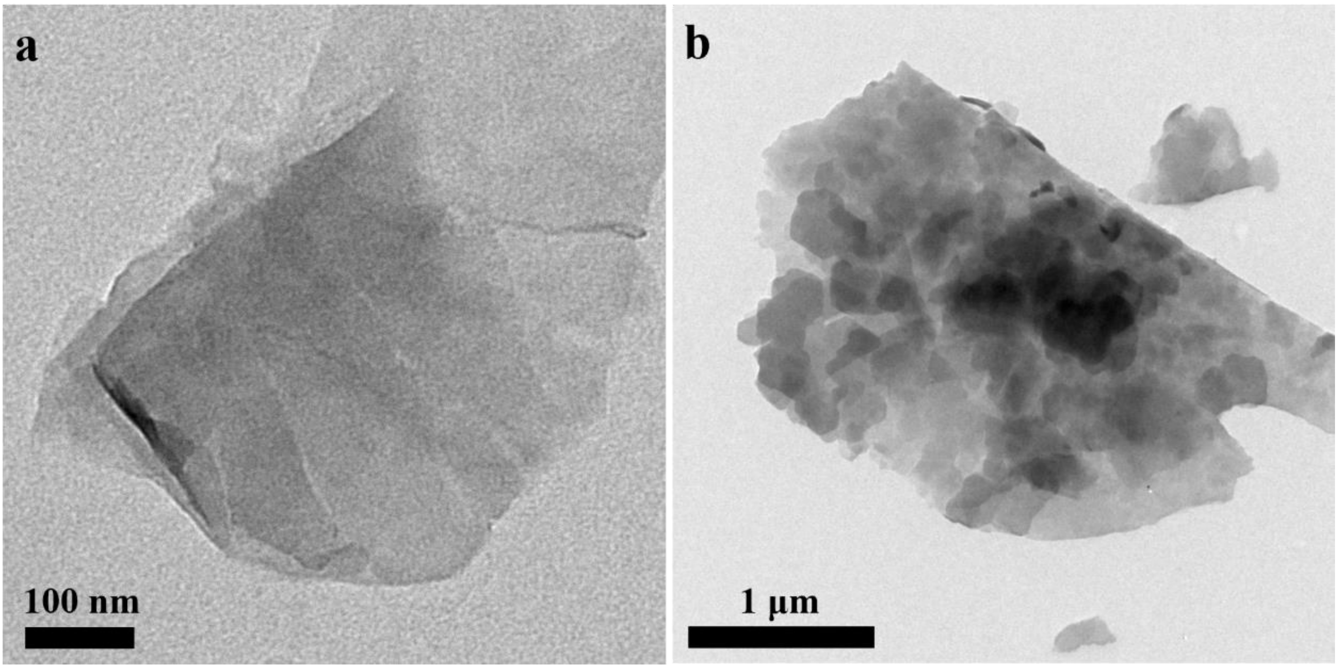

Supplementary Figure 17. TEM images of (a) as-synthesized Zr-BTB-PABA nanosheets and (b) untwisted Zr-BTB-PABA nanosheets after heating.

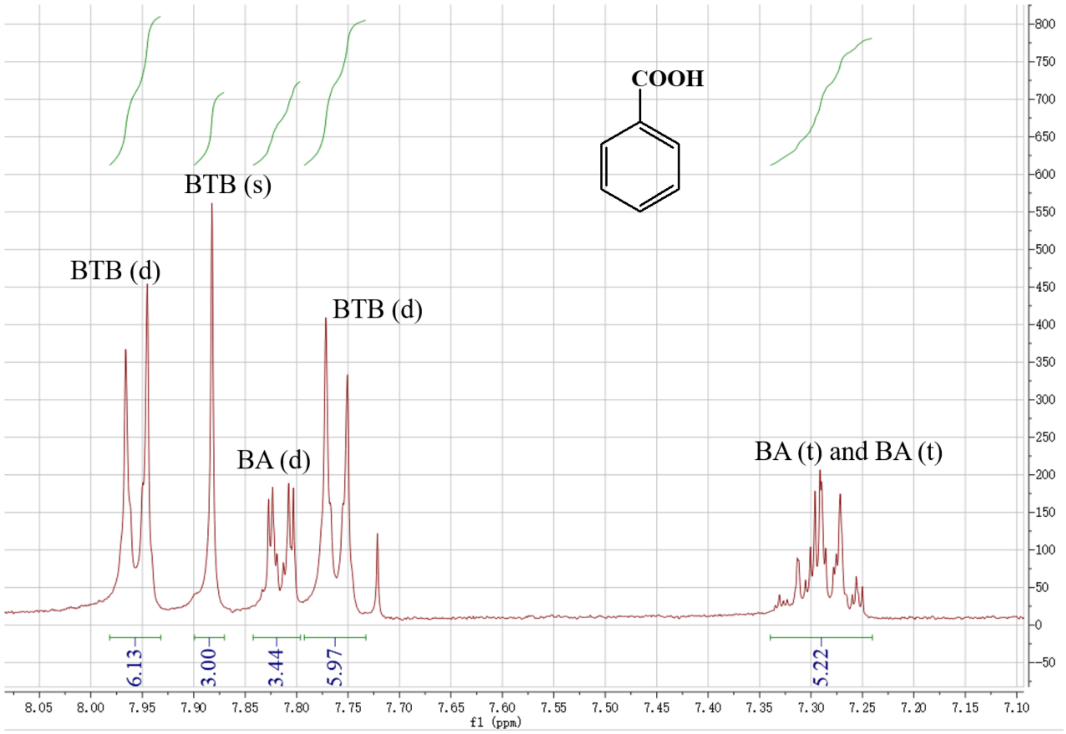

Supplementary Figure 18. H^1NMR of 2-D Zr-BTB-BA nanosheets. Molar ratio of n(BA): n(BTB)=1.72: 1 was obtained from the peak integration. The nanosheets sample (around 5 mg) was digested in d_6-DMSO/ K_3PO_4-D_2O (4:1) before H^1NMR measurement.

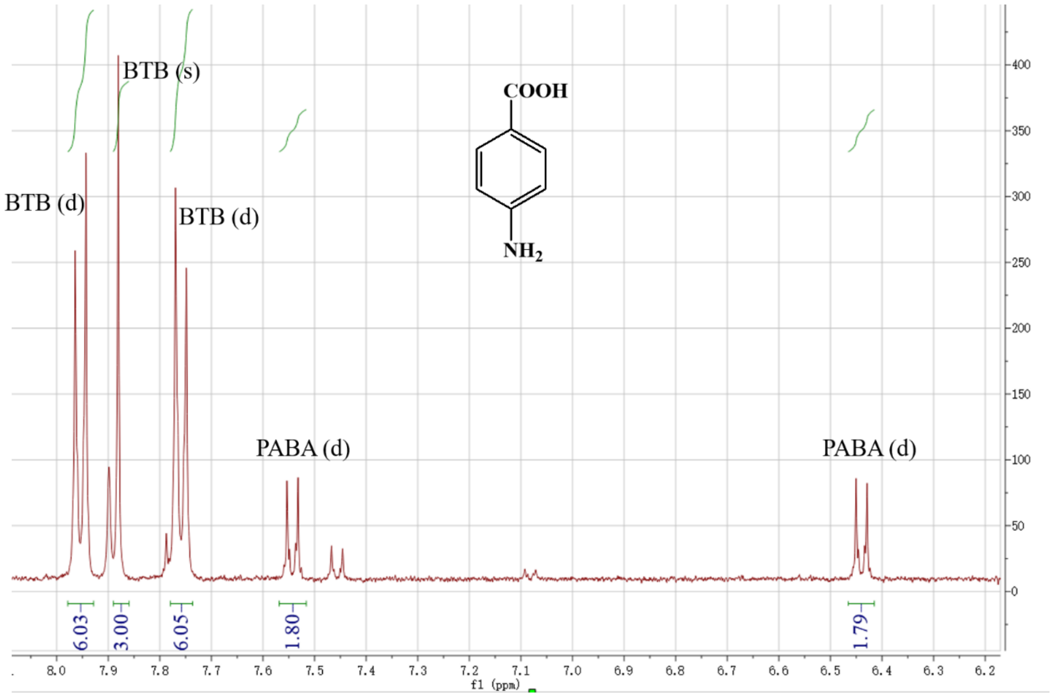

Supplementary Figure 19. H^1NMR of 2-D Zr-BTB-PABA nanosheets. Molar ratio of n(PABA): n(BTB)=0.9: 1 was obtained from the peak integration. The nanosheets sample (around 5 mg) was digested in d_6-DMSO/ K_3PO_4-D_2O (4:1) before H^1NMR measurement.

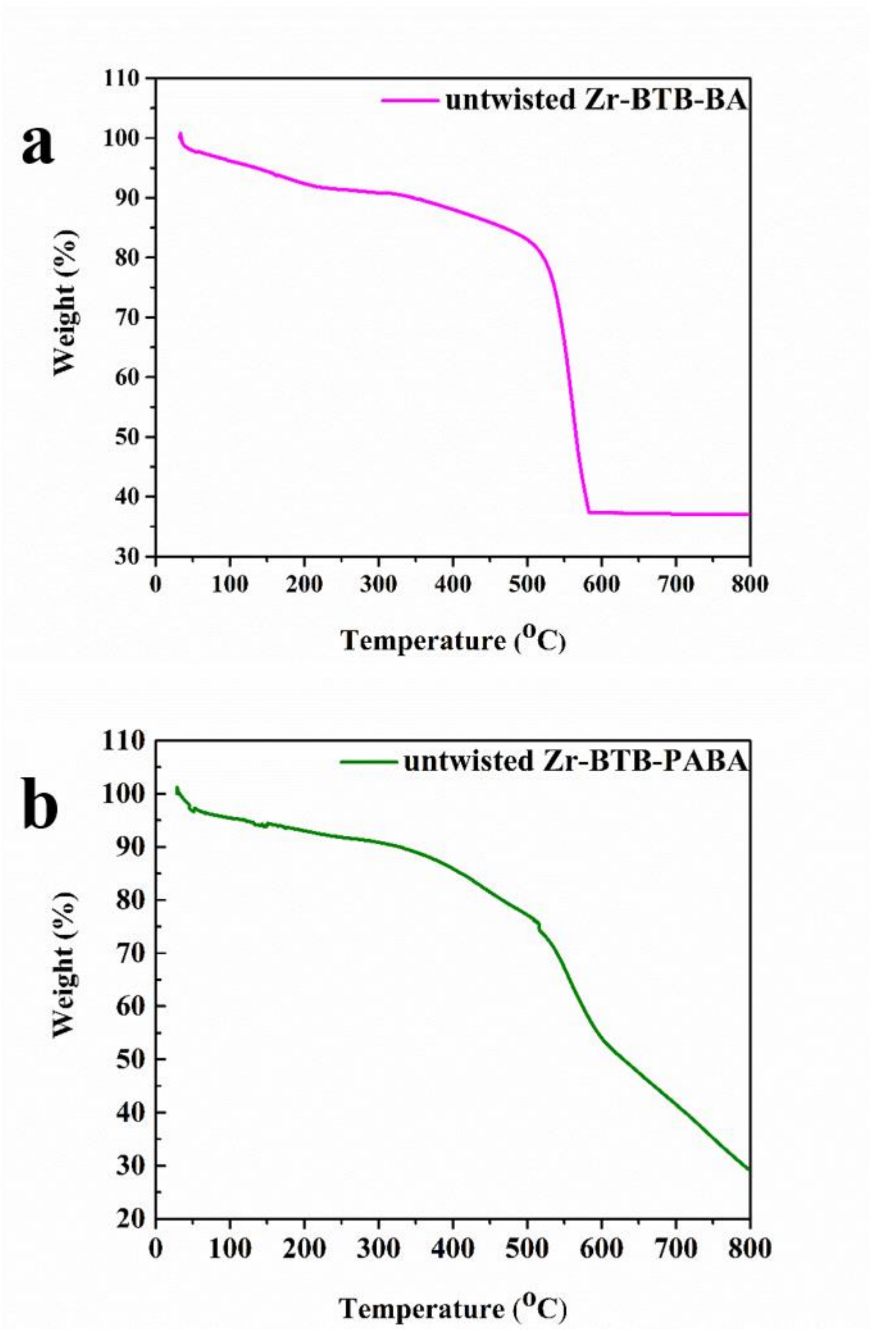

Supplementary Figure 20. TGA curves of the as-synthesized 2-D (a) untwisted Zr-BTB-BA, (b) untwisted Zr-BTB-PABA. Experiment condition: temperature ramp from room temperature to 800 ºC at 10 ºC·min^{-1} under N_2 flow.

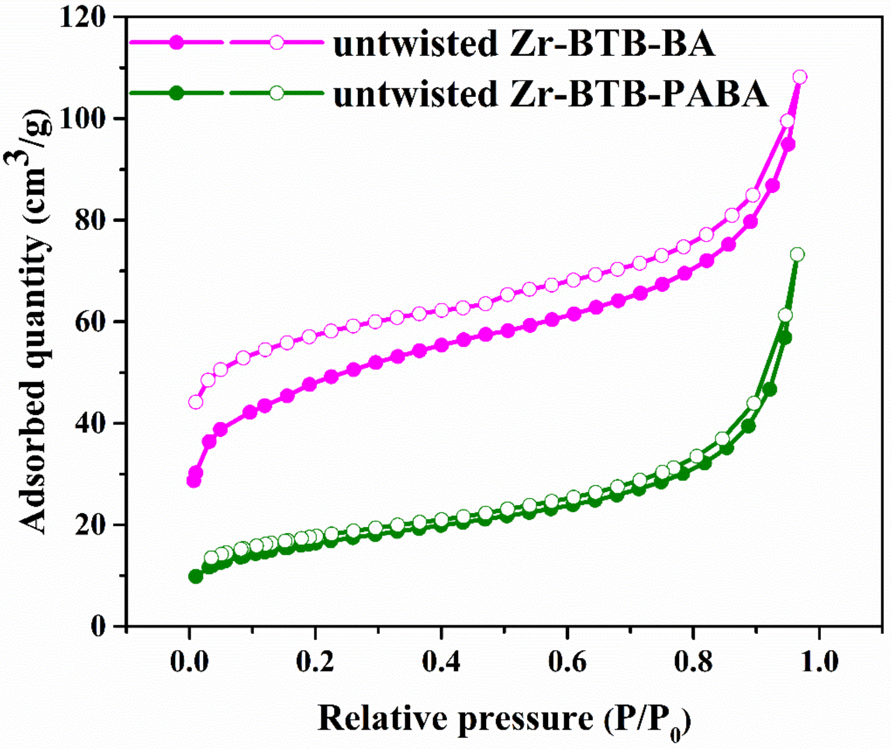

Supplementary Figure 21. N_2 adsorption-desorption isotherms of 2-D untwisted Zr-BTB-BA and untwisted Zr-BTB-PABA nanosheets. The Brunauer-Emmett-Teller (BET) surface and the total pore volumes of as-synthesized untwisted Zr-BTB-BA and Zr-BTB-PABA nanosheets were 160.9 $m^2 \cdot g^{-1}$ 0.116 $cm^3 \cdot g^{-1}$ and 56.6 $m^2 \cdot g^{-1}$ 0.083 $cm^3 \cdot g^{-1}$, respectively.

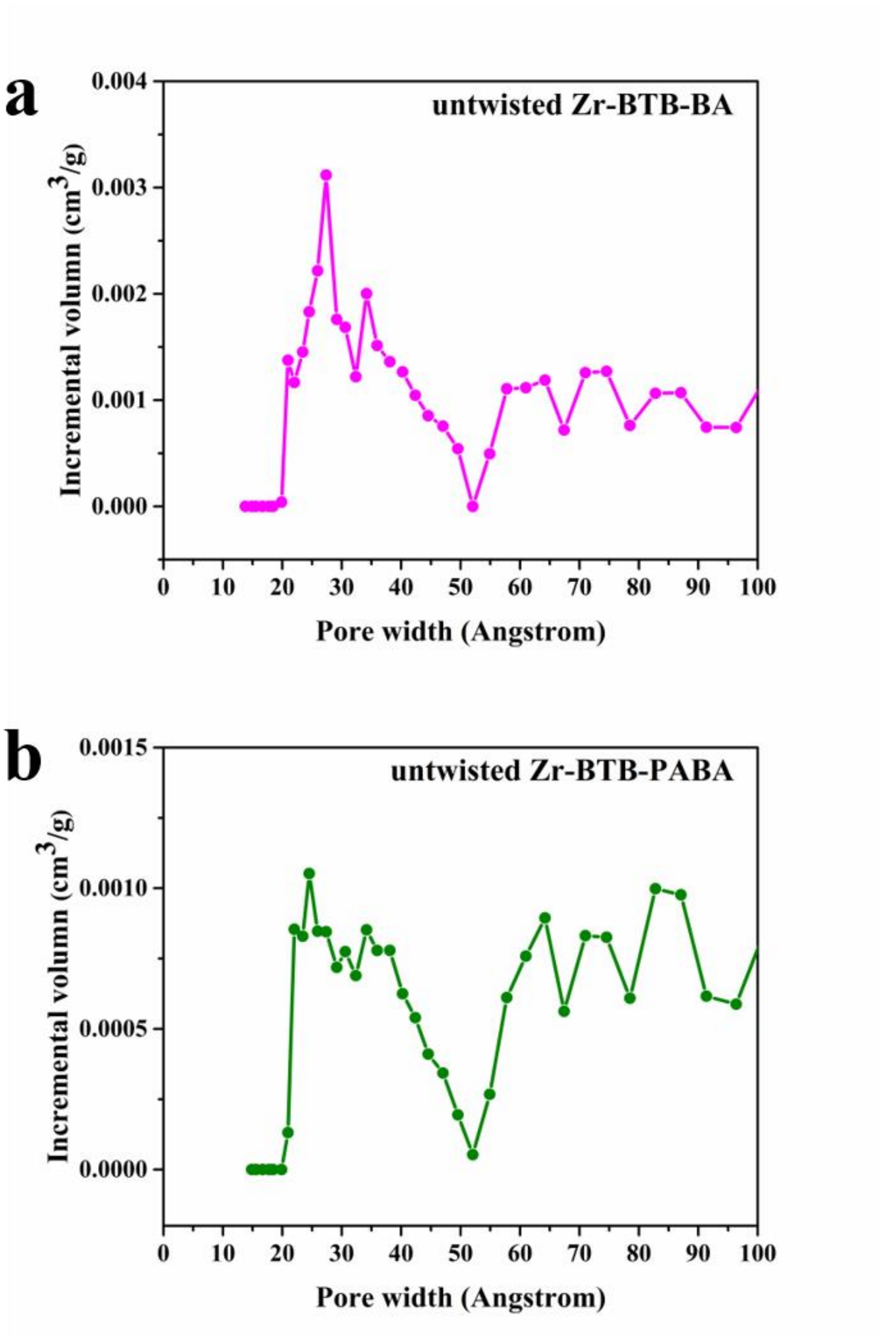

Supplementary Figure 22. Pore size distributions calculated via NLDFT method for (a) untwisted Zr-BTB-BA nanosheets and (b) untwisted Zr-BTB-PABA nanosheets.

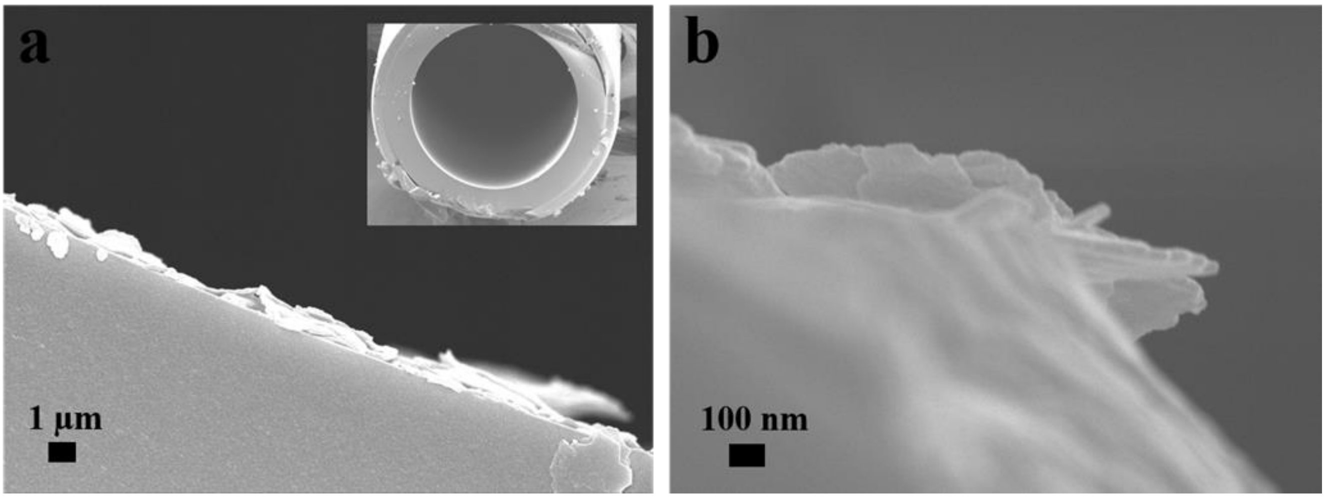

Supplementary Figure 23. SEM images of (a) 2-D twisted Zr-BTB-FA nanosheets coated capillary column (0.9 µm thickness) with cross-section view; (b) 2-D twisted Zr-BTB-FA nanosheets on the inner wall of capillary column. Taking of stationary thickness into consideration and make a fair comparison, the column was coated with 2 times.

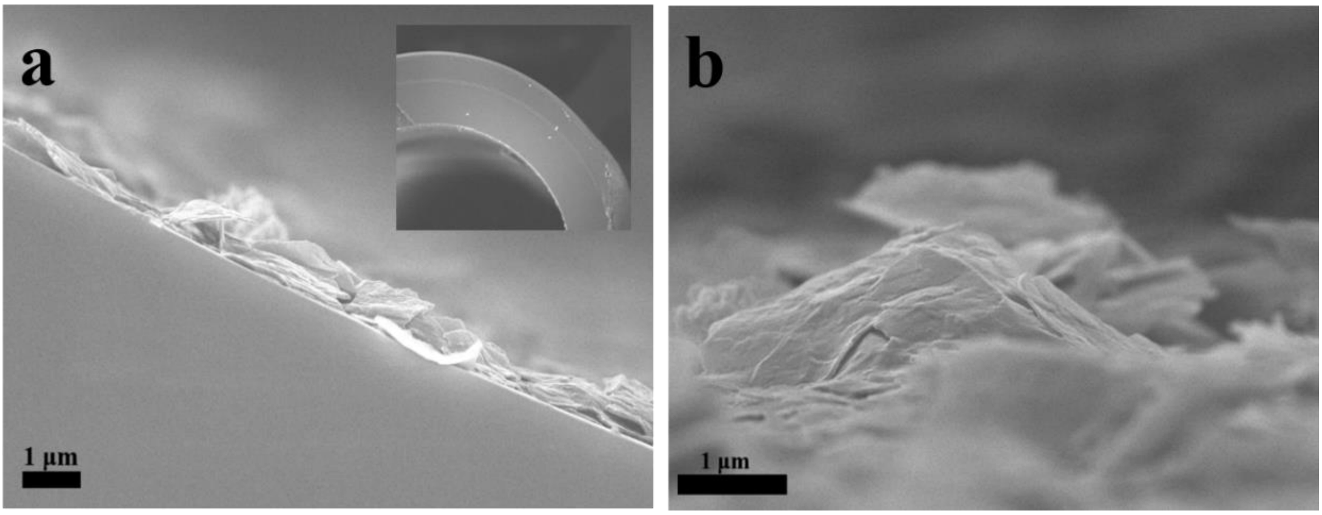

Supplementary Figure 24. SEM images of (a) 2-D untwisted Zr-BTB-FA nanosheets coated capillary column (0.9 µm thickness) with cross-section view; (b) 2-D untwisted Zr-BTB-FA nanosheets on the inner wall of capillary column.

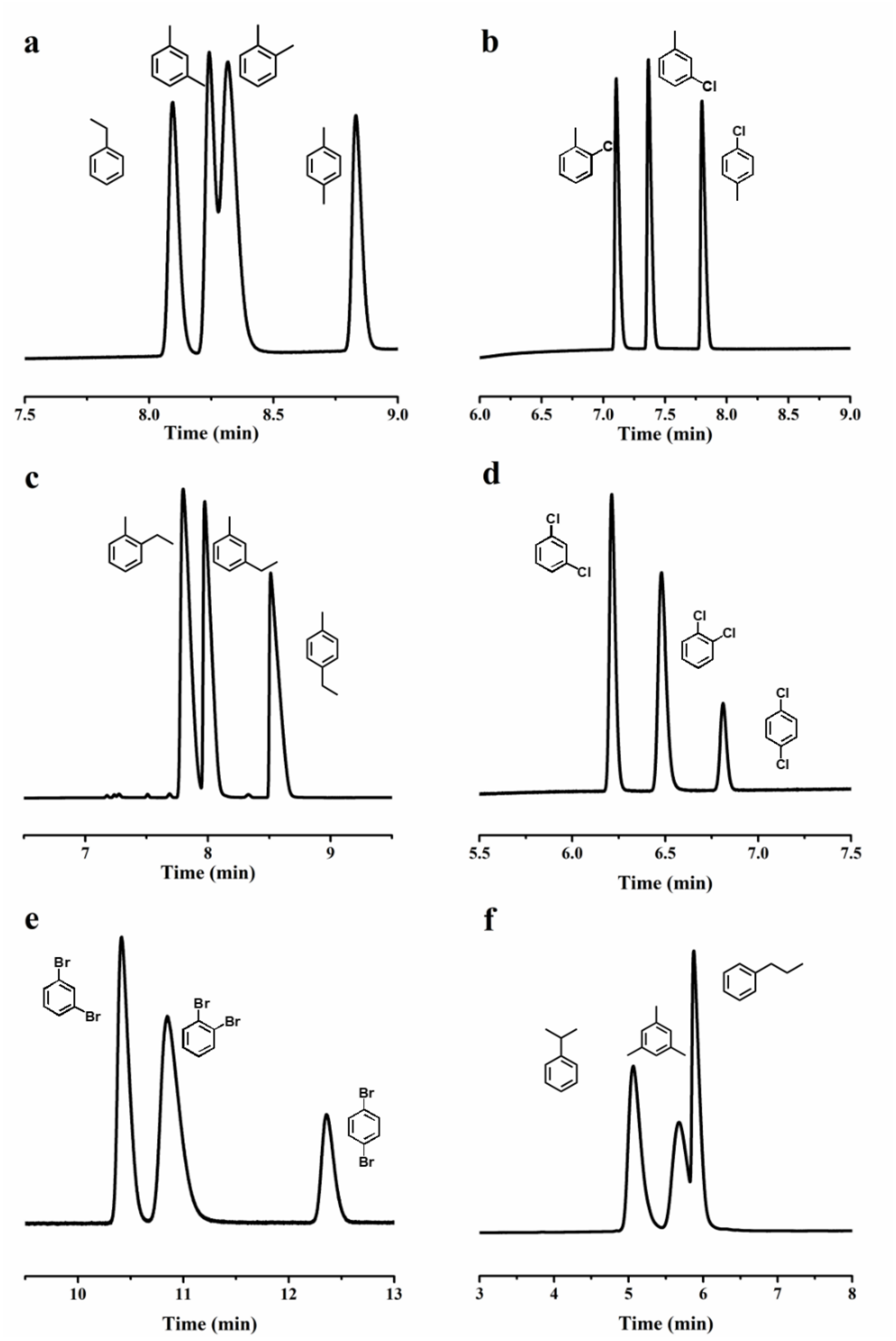

Supplementary Figure 25. Gas chromatograms on the 2-D untwisted Zr-BTB-FA capillary column (30 m long × 0.25 mm i.d., 0.9 μm thickness) under N_2 flow of 1.0 mL·min^{-1} for separation of: (a) xylene isomers and ethylbenzene using a temperature program of 160 ºC for 1 min, and then 10 ºC·min^{-1} to 250 ºC; (b) chlorotoluene isomers using a temperature program of 150 ºC for 1 min, and then 20 ºC·min^{-1} to 250 ºC; (c) ethyltoluene isomers using a temperature program of 100 ºC for 1 min, and then 30 ºC·min^{-1} to 250 ºC; (d) dichlorobenzene isomers using a temperature program of 180 ºC for 1 min, and then 20 ºC·min^{-1} to 250 ºC; (e) dibromobenzene isomers using a temperature program of 180 ºC for 1 min, and then 20 ºC·min^{-1} to 250 ºC; (f) mixtures of n-propylbenzene, isopropylbenzene and 1,3,5-trimethylbenzene using a temperature program of 180 ºC for 1 min, and then 20 ºC·min^{-1} to 250 ºC.

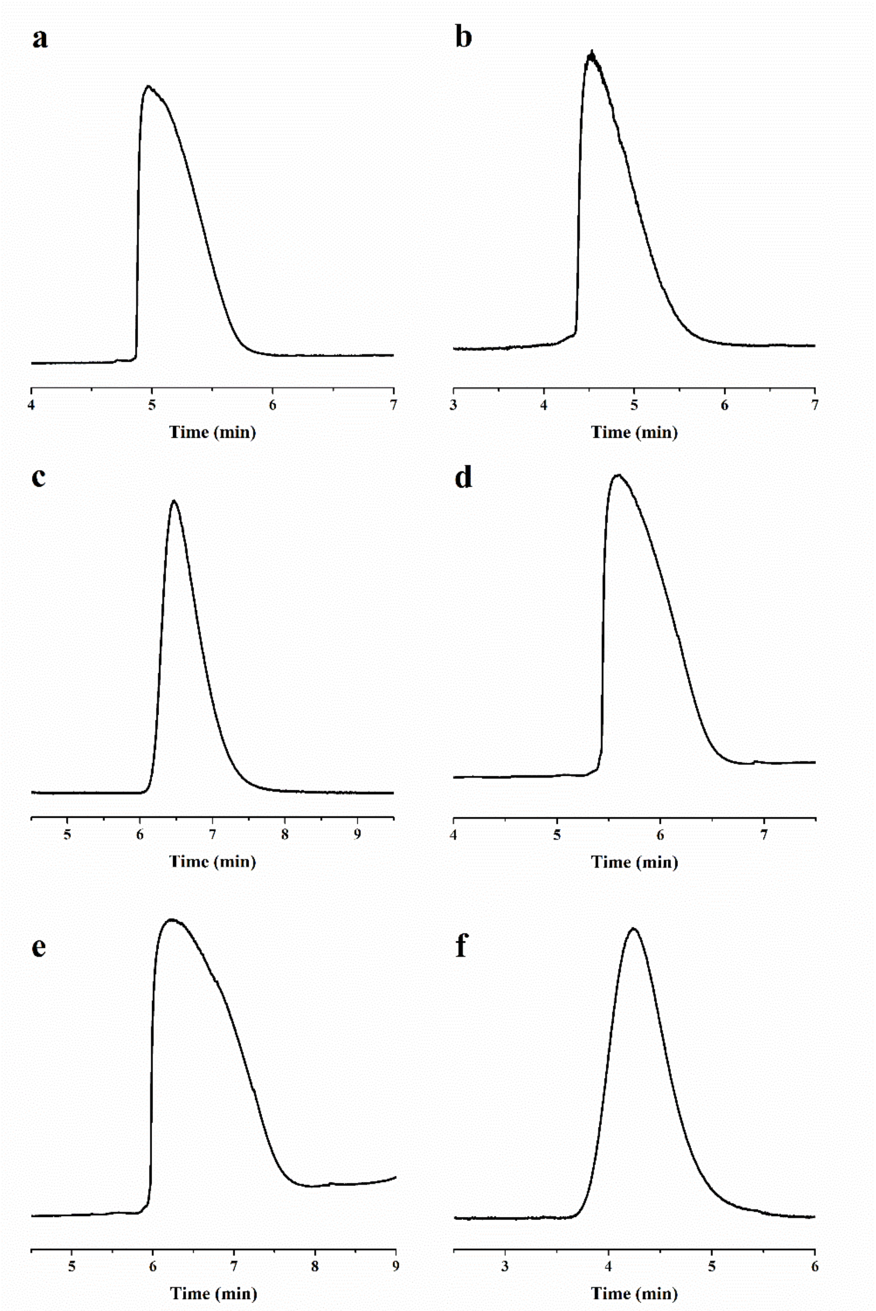

Supplementary Figure 26. Gas chromatograms on the 2-D twisted Zr-BTB-FA capillary column (30 m long × 0.25 mm i.d., 0.9 μm thickness) under N_2 flow of 1.0 mL·min^{-1} for separation of: (a) xylene isomers and ethylbenzene using a temperature program of 100 °C for 1 min, and then 20 °C·min^{-1} to 220 °C; (b) chlorotoluene isomers using a temperature program of 100 °C for 1 min, and then 20 °C·min^{-1} to 200 °C; (c) ethyltoluene isomers using a temperature program of 100 °C for 1 min, and then 20 °C·min^{-1} to 200 °C; (d) dichlorobenzene isomers using a temperature program of 100 °C for 1 min, and then 20 °C·min^{-1} to 220 °C; (e) dibromobenzene isomers using a temperature program of 100 °C for 1 min, and then 20 °C·min^{-1} to 220 °C; (f) mixtures of n-propylbenzene, isopropylbenzene and 1,3,5-trimethylbenzene using a temperature program of 100 °C for 1 min, and then 20 °C·min^{-1} to 220 °C.

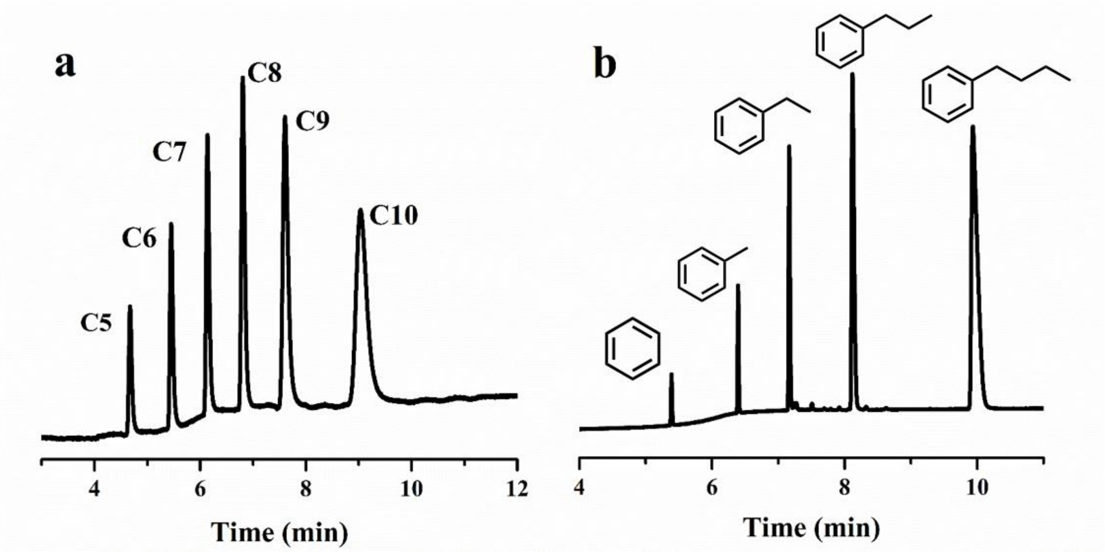

Supplementary Figure 27. Gas chromatograms on the 2-D untwisted Zr-BTB-FA coated capillary column (30 m long × 0.25 mm i.d.) at N_2 flow rate of 1 mL·min^{-1} for separation of: (a) linear alkanes using a temperature program of 60 ºC for 1 min, and then 40 ºC·min^{-1} to 250 ºC; (b) benzene homologues using a temperature program of 100 ºC for 1 min, and then 30 ºC·min^{-1} to 250 ºC.

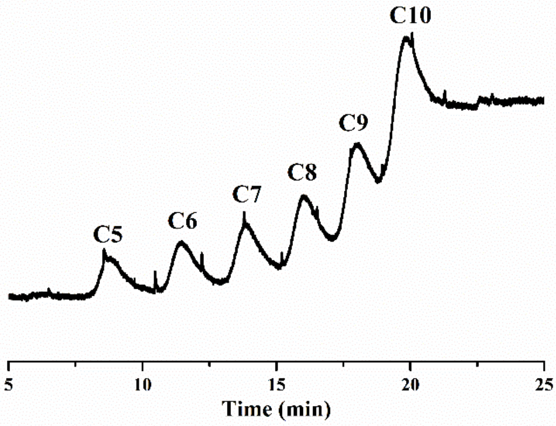

Supplementary Figure 28. Gas chromatograms on the 2-D twisted Zr-BTB-FA nanosheets coated capillary column (30 m long × 0.25 mm i.d.) under N_2 flow of 1.0mL·min^{-1} for separation of linear alkanes using a temperature program of 60 ºC for 1 min, and then 10 ºC·min^{-1} to 250 ºC.

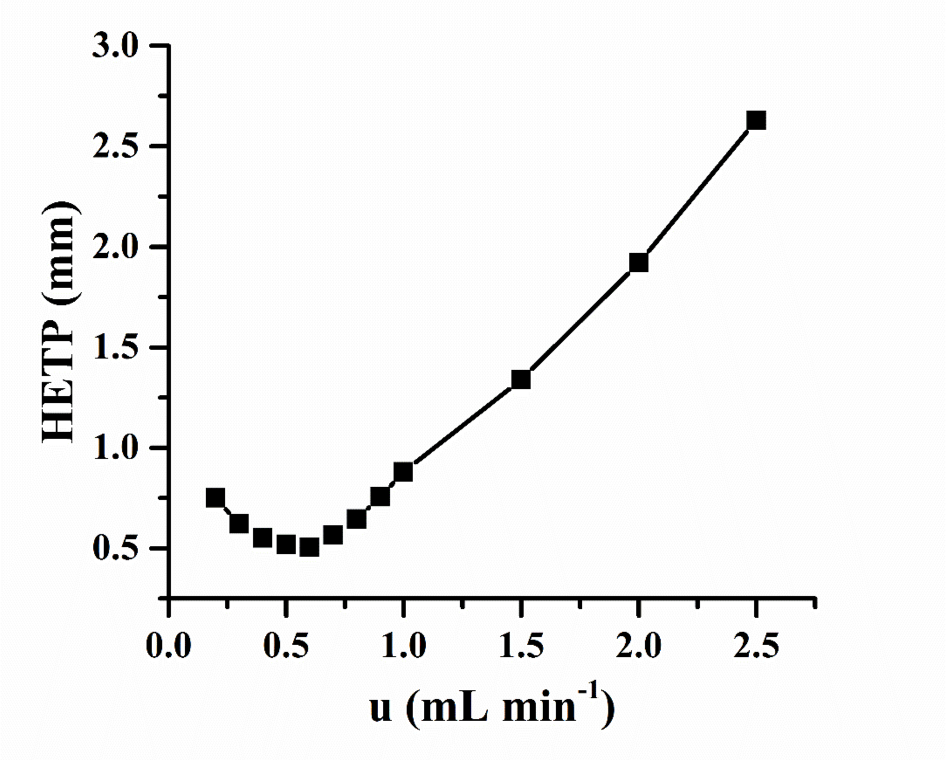

Supplementary Figure 29. van Deemter curve of the 2-D untwisted Zr-BTB-FA nanosheets coated capillary column determined by p-chlorotoluene at 250 °C.

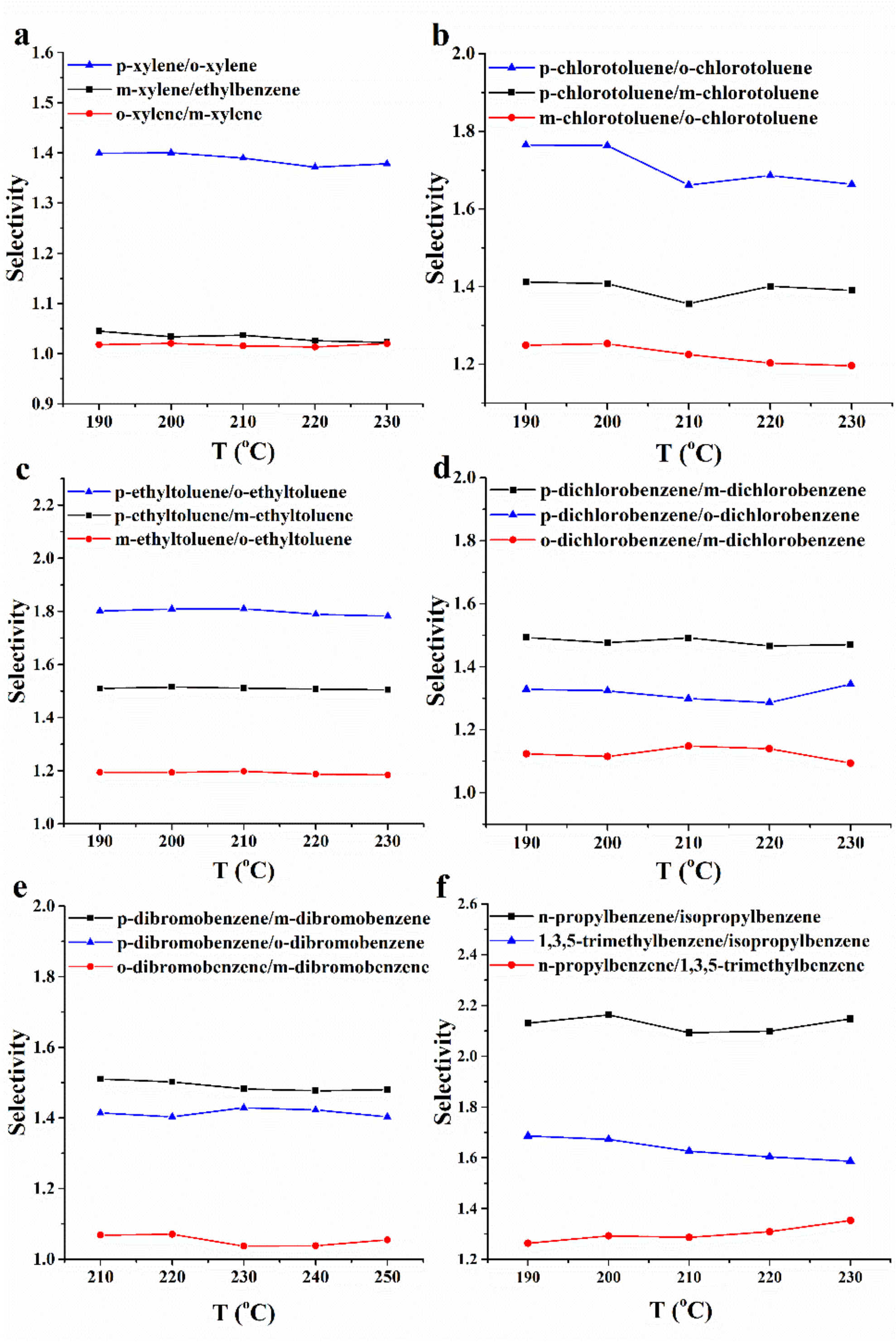

Supplementary Figure 30. Effect of temperature on the selectivity for the separation of structural isomers of benzene derivatives on the 2-D untwisted Zr-BTB-FA nanosheets coated capillary column: (a) xylene isomers and ethylbenzene; (b) chlorotoluene isomers; (c) ethyltoluene isomers; (d) dichlorobenzene isomers; (e) dibromobenzene isomers; (f) n-propylbenzene, isopropylbenzene and 1,3,5-trimethylbenzene.

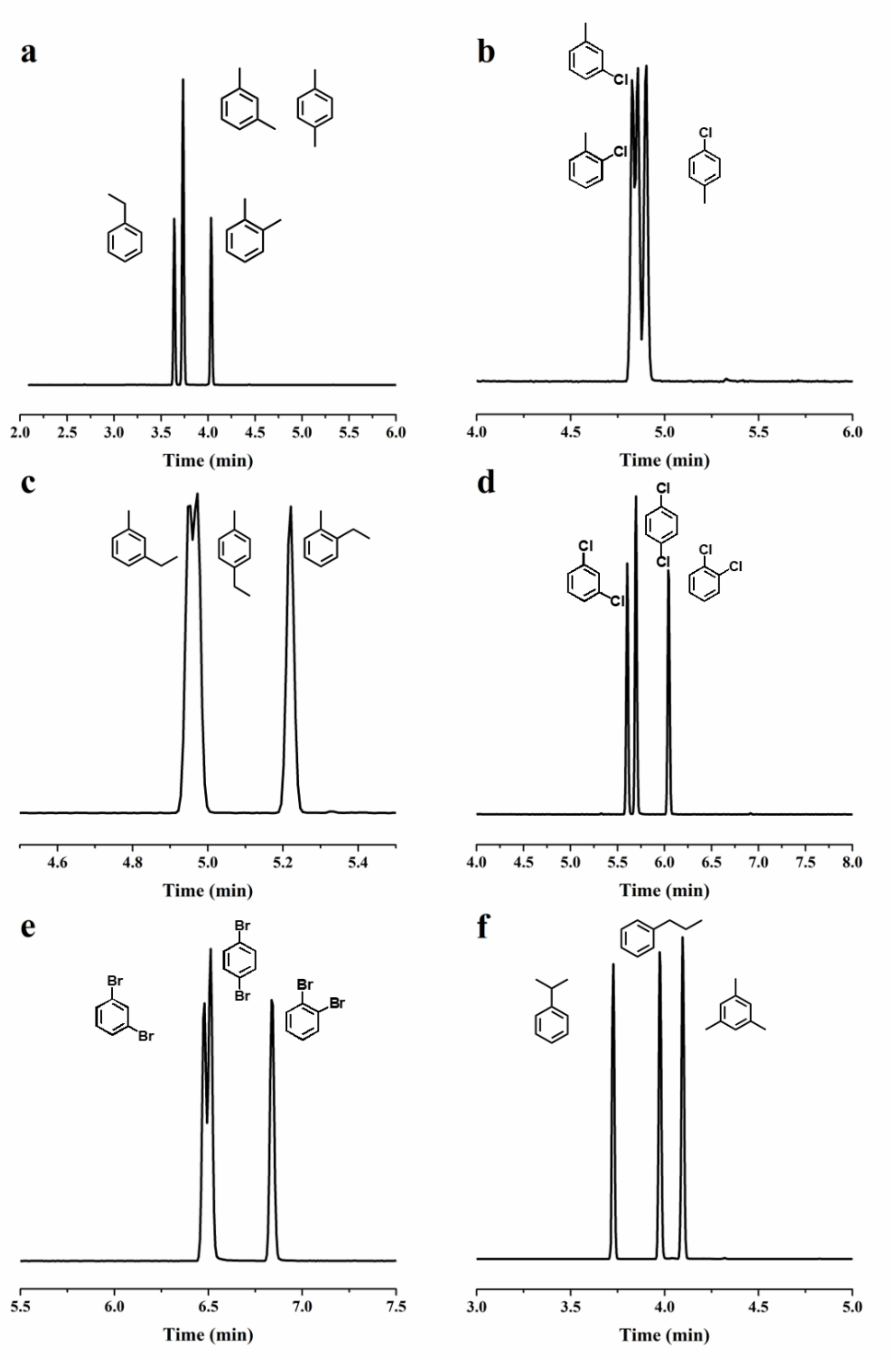

Supplementary Figure 31. Gas chromatograms on the commercial HP-5MS capillary column (30 m long × 0.25 mm i.d.) under He flow of 1.0 mL·min^{-1} for separation of : (a) xylene isomers and ethylbenzene using a temperature program of 60 ºC for 1 min, and then 10 ºC·min^{-1} to 180 ºC; (b) chlorotoluene isomers using a temperature program of 60 ºC for 1 min, and then 10 ºC·min^{-1} to 180 ºC; (c) ethyltoluene isomers using a temperature program of 60 ºC for 1 min, and then 10 ºC·min^{-1} to 160 ºC; (d) dichlorobenzene isomers using a temperature program of 60 ºC for 1 min, and then 10 ºC·min^{-1} to 180 ºC; (e) dibromobenzene isomers using a temperature program of 80 ºC for 1 min, and then 10 ºC·min^{-1} to 160 ºC; (f) mixtures of n-propybenzene, isopropybenzene and 1,3,5-trimethylbenzene using a temperature program of 60 ºC for 1 min, and then 20 ºC·min^{-1} to 230 ºC. The experiments were conducted on an Agilent 5977A system equipped with a mass spectrometry detector.

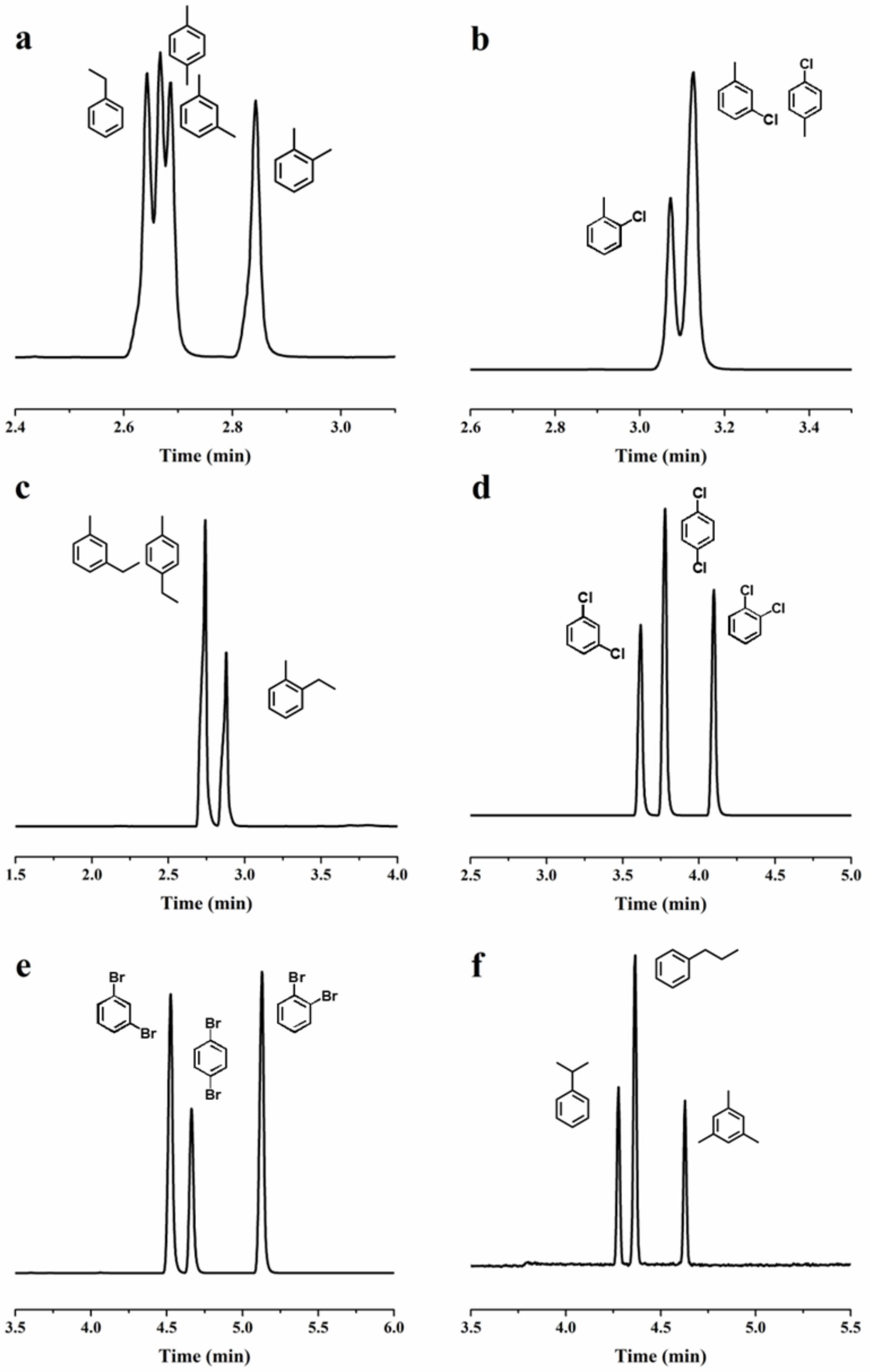

Supplementary Figure 32. Gas chromatograms on the commercial VF-WAXMS capillary column (30 m long × 0.25 mm i.d.) under N_2 flow of 1.0 mL·min^{-1} for separation of: (a) xylene isomers and ethylbenzene using a temperature program of 120 ºC for 1 min, and then 10 ºC·min^{-1} to 200 ºC; (b) chlorotoluene isomers using a temperature program of 130 ºC for 1 min, and then 10 ºC·min^{-1} to 200 ºC; (c) ethyltoluene isomers using a temperature program of 130 ºC for 1 min, and then 10 ºC·min^{-1} to 200 ºC; (d) dichlorobenzene isomers using a temperature program of 130 ºC for 1 min, and then 10 ºC·min^{-1} to 200 ºC; (e) dibromobenzene isomers using a temperature program of 150 ºC for 1 min, and then 10 ºC·min^{-1} to 220 ºC; (f) mixtures of n-propybenzene, isopropybenzene and 1,3,5-trimethylbenzene using a temperature program of 60 ºC for 1 min, and then 20 ºC·min^{-1} to 200 ºC.

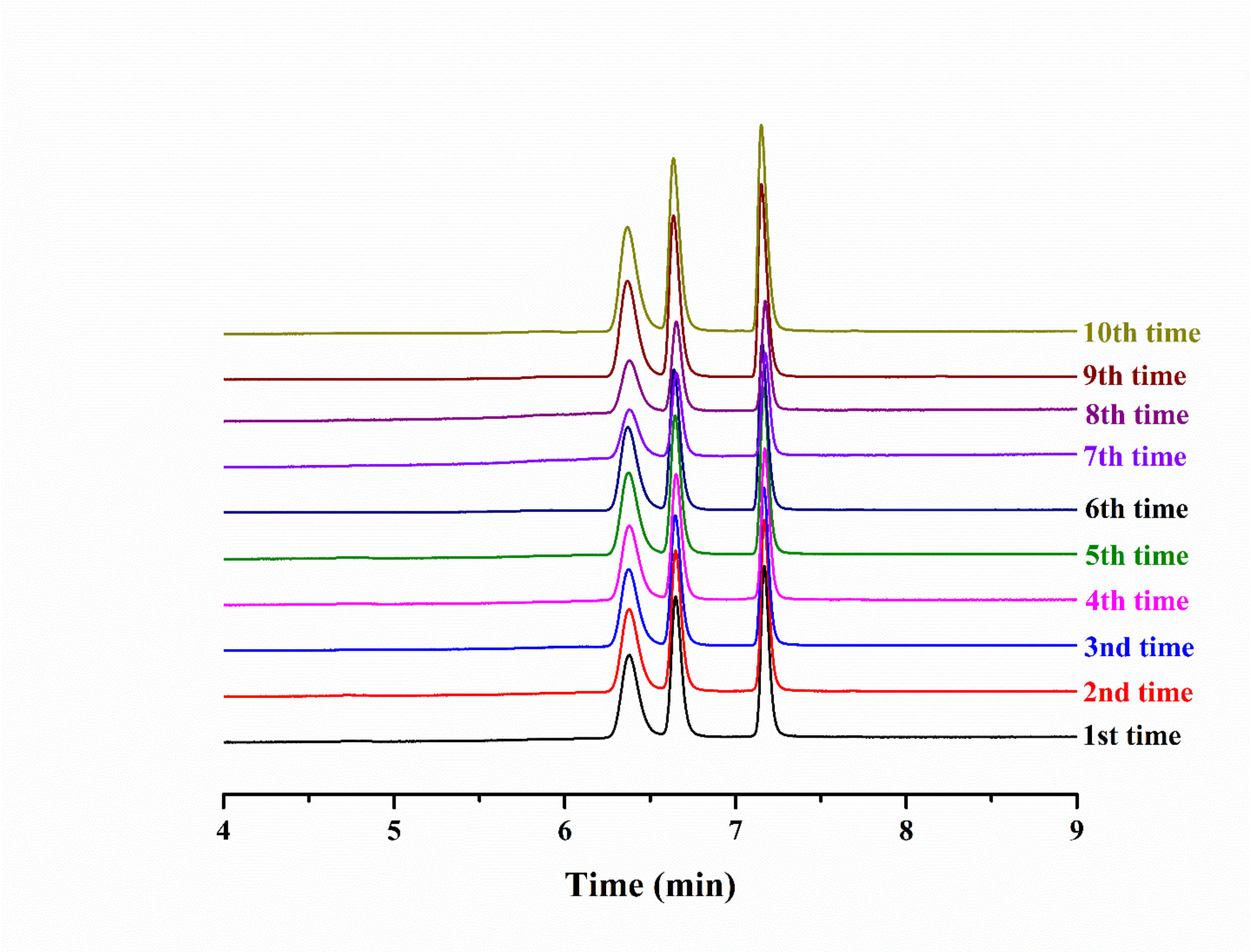

Supplementary Figure 33. The thermal stability and repeatability of 2-D untwisted Zr-BTB-FA capillary column for separation of chlorotoluene isomers 10 times continuously. After every separation experiment, the column was heated to 250 °C for 30 min.

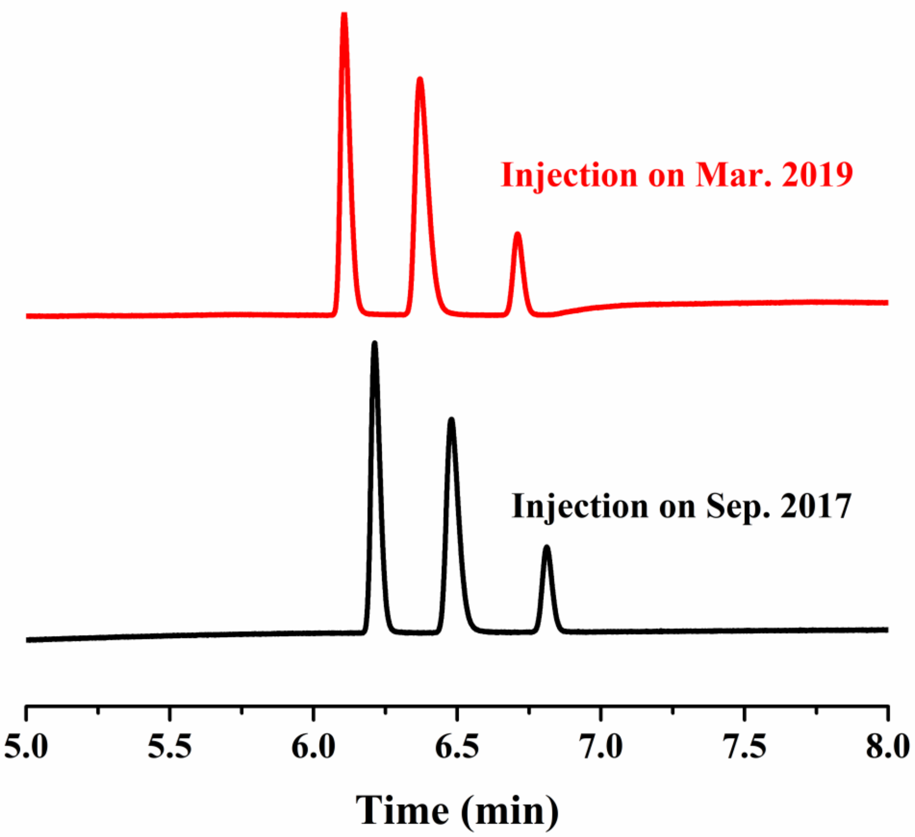

Supplementary Figure 34. The lifetime of 2-D untwisted Zr-BTB-FA capillary column for separation of dichlorobenzene isomers under the same experimental condition (a temperature program of 180 ºC for 1 min, and then 20 ºC·min^{-1} to 250 ºC) within 16 months. The separation efficiency and peak shape did not changed. The drift of retention time was attributed to shortening of capillary column length due to the unavoidable depletion.

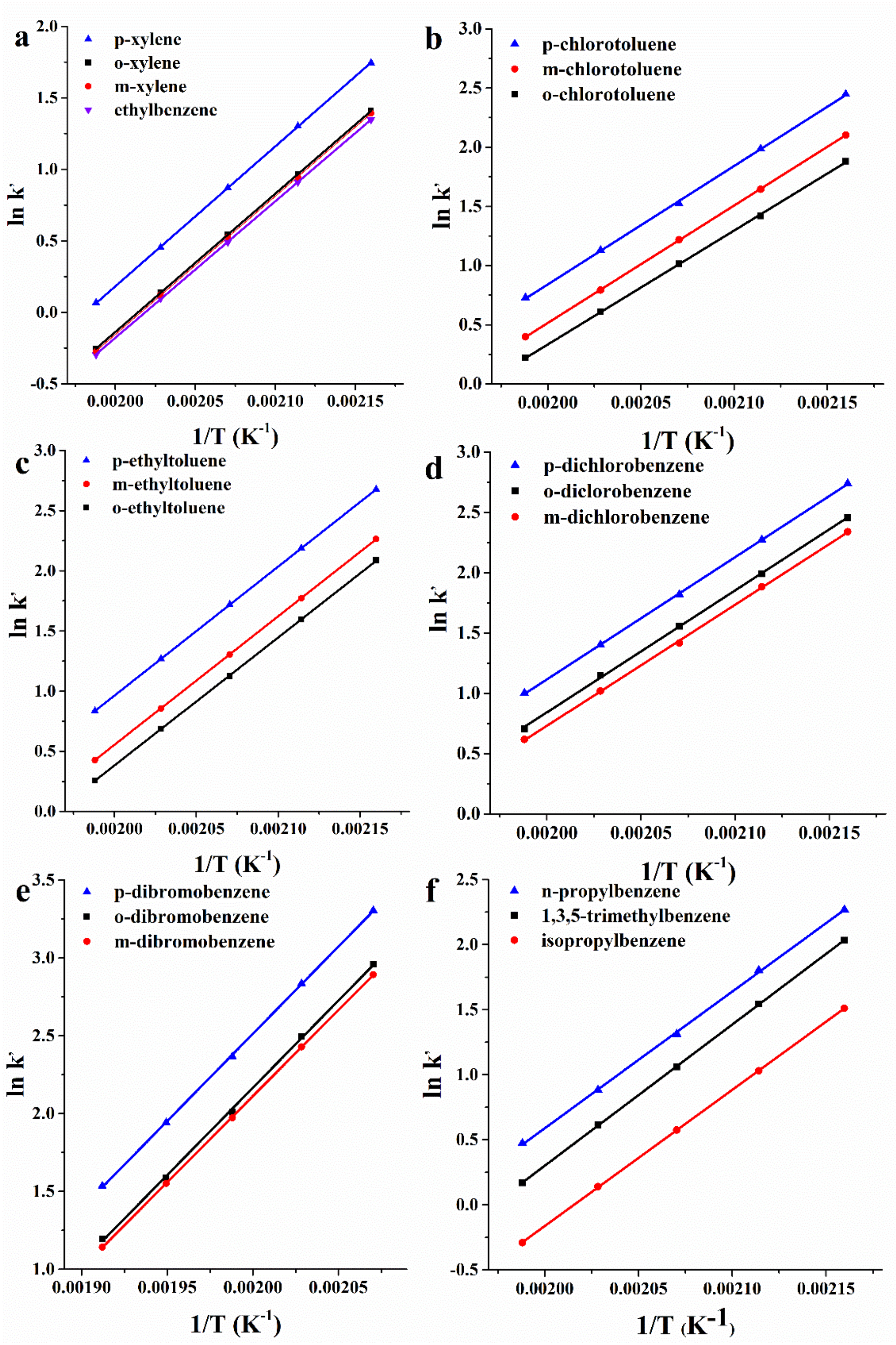

Supplementary Figure 35. van't Hoff plots for the GC separation of structural isomers of benzene derivatives on the 2-D untwisted Zr-BTB-FA nanosheets coated capillary column.

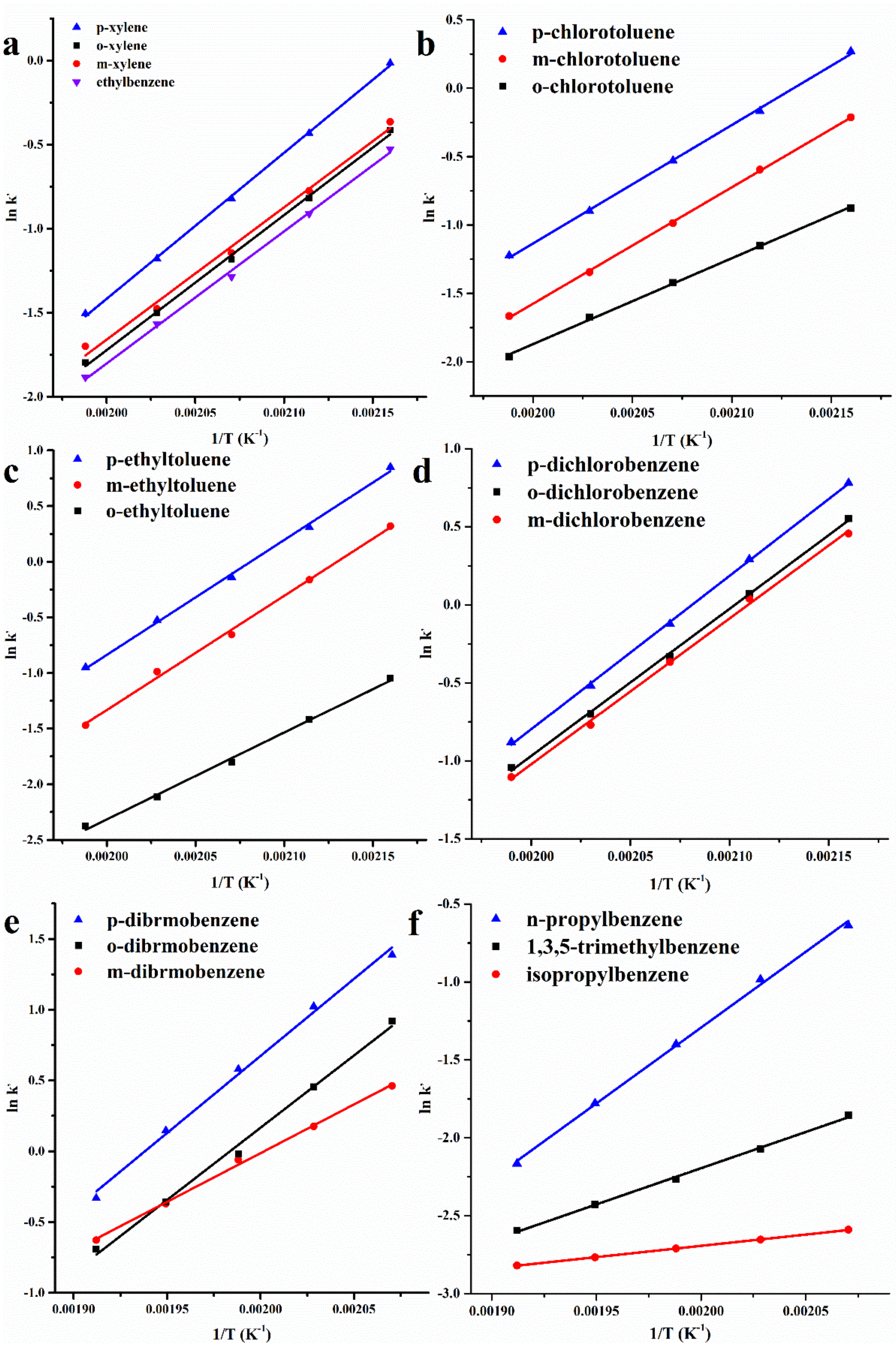

Supplementary Figure 36. van't Hoff plots for the GC separation of structural isomers of benzene derivatives on the 2-D twisted Zr-BTB-FA nanosheets coated capillary column.

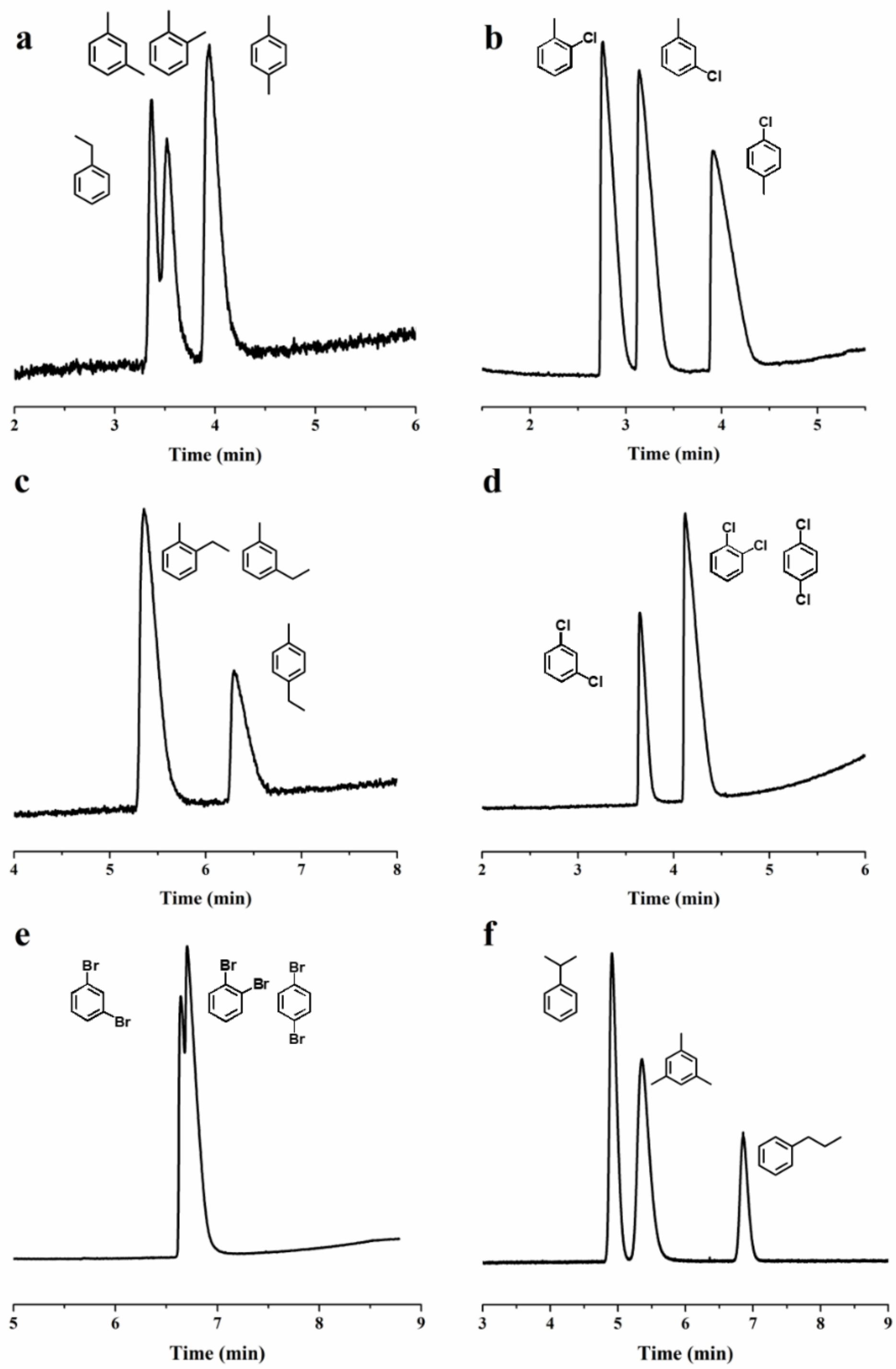

Supplementary Figure 37. Gas chromatograms on the 2-D untwisted Zr-BTB-BA capillary column (30 m long × 0.25 mm i.d.) under N_2 flow of 1.0 mL·min^{-1} for separation of: (a) xylene isomers and ethylbenzene using a temperature program of 80 ºC for 1 min, and then 20 ºC·min^{-1} to 200 ºC; (b) chlorotoluene isomers using a temperature program of 100 ºC for 1 min, and then 10 ºC·min^{-1} to 200 ºC; (c) ethyltoluene isomers using a temperature program of 80 ºC for 1 min, and then 10 ºC·min^{-1} to 200 ºC; (d) dichlorobenzene isomers using a temperature program of 80 ºC for 1 min, and then 20 ºC·min^{-1} to 200 ºC; (e) dibromobenzene isomers using a temperature program of 120 ºC for 1 min, and then 20 ºC·min^{-1} to 250 ºC; (f) mixtures of n-propylbenzene, isopropylbenzene and 1,3,5-trimethylbenzene using a temperature program of 100 ºC for 1 min, and then 20 ºC·min^{-1} to 250 ºC.

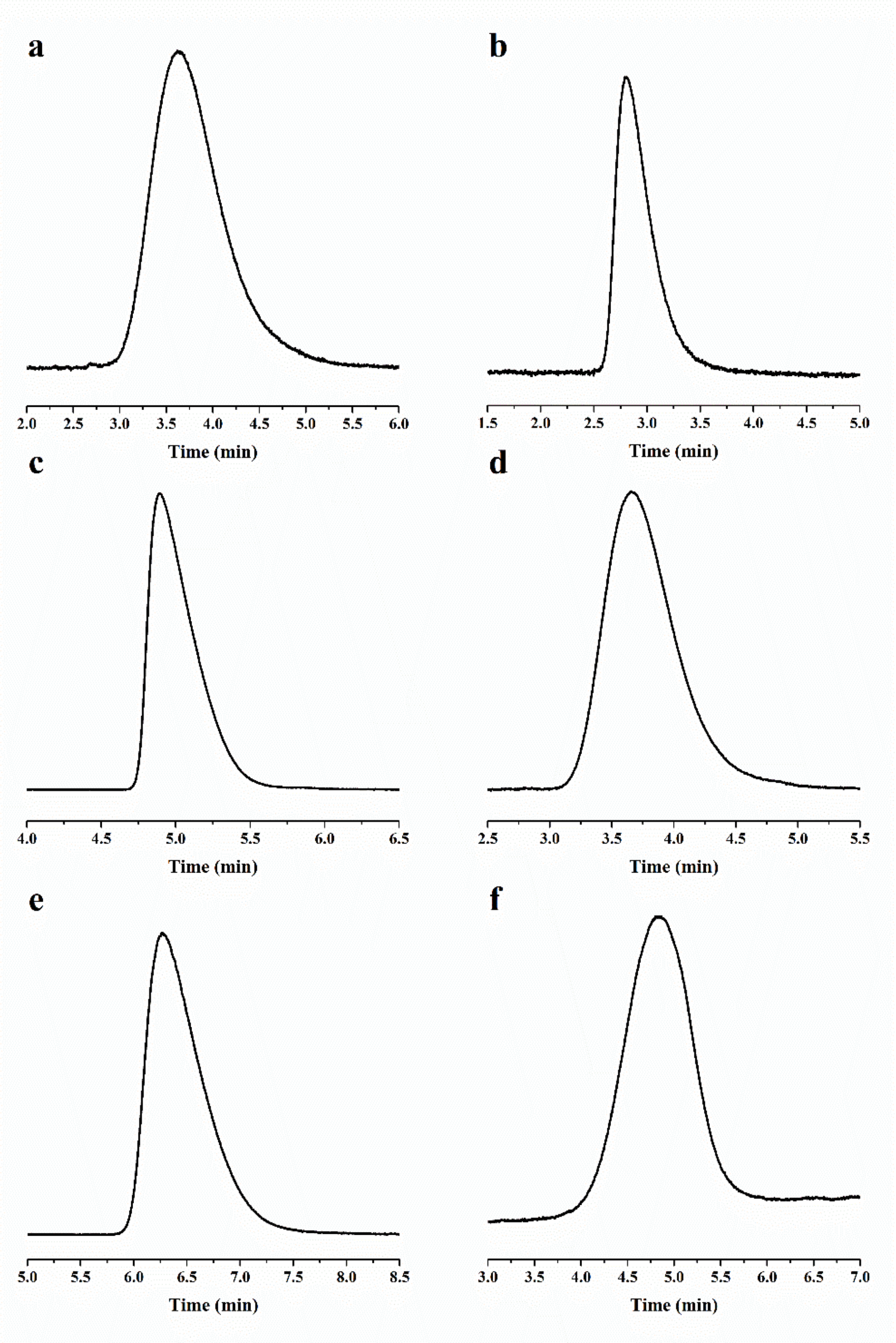

Supplementary Figure 38. Gas chromatograms on the 2-D twisted Zr-BTB-BA capillary column (30 m long × 0.25 mm i.d.) under N_2 flow of 1.0 mL·min^{-1} for separation of: (a) xylene isomers and ethylbenzene using a temperature program of 80 °C for 1 min, and then 20 °C·min^{-1} to 200 °C; (b) chlorotoluene isomers using a temperature program of 80 °C for 1 min, and then 10 °C·min^{-1} to 200 °C; (c) ethyltoluene isomers using a temperature program of 80 °C for 1 min, and then 20 °C·min^{-1} to 200 °C; (d) dichlorobenzene isomers using a temperature program of 80 °C for 1 min, and then 20 °C·min^{-1} to 200 °C; (e) dibromobenzene isomers using a temperature program of 120 °C for 1 min, and then 20 °C·min^{-1} to 250 °C; (f) mixtures of n-propylbenzene, isopropylbenzene and 1,3,5-trimethylbenzene using a temperature program of 100 °C for 1 min, and then 20 °C·min^{-1} to 250 °C.

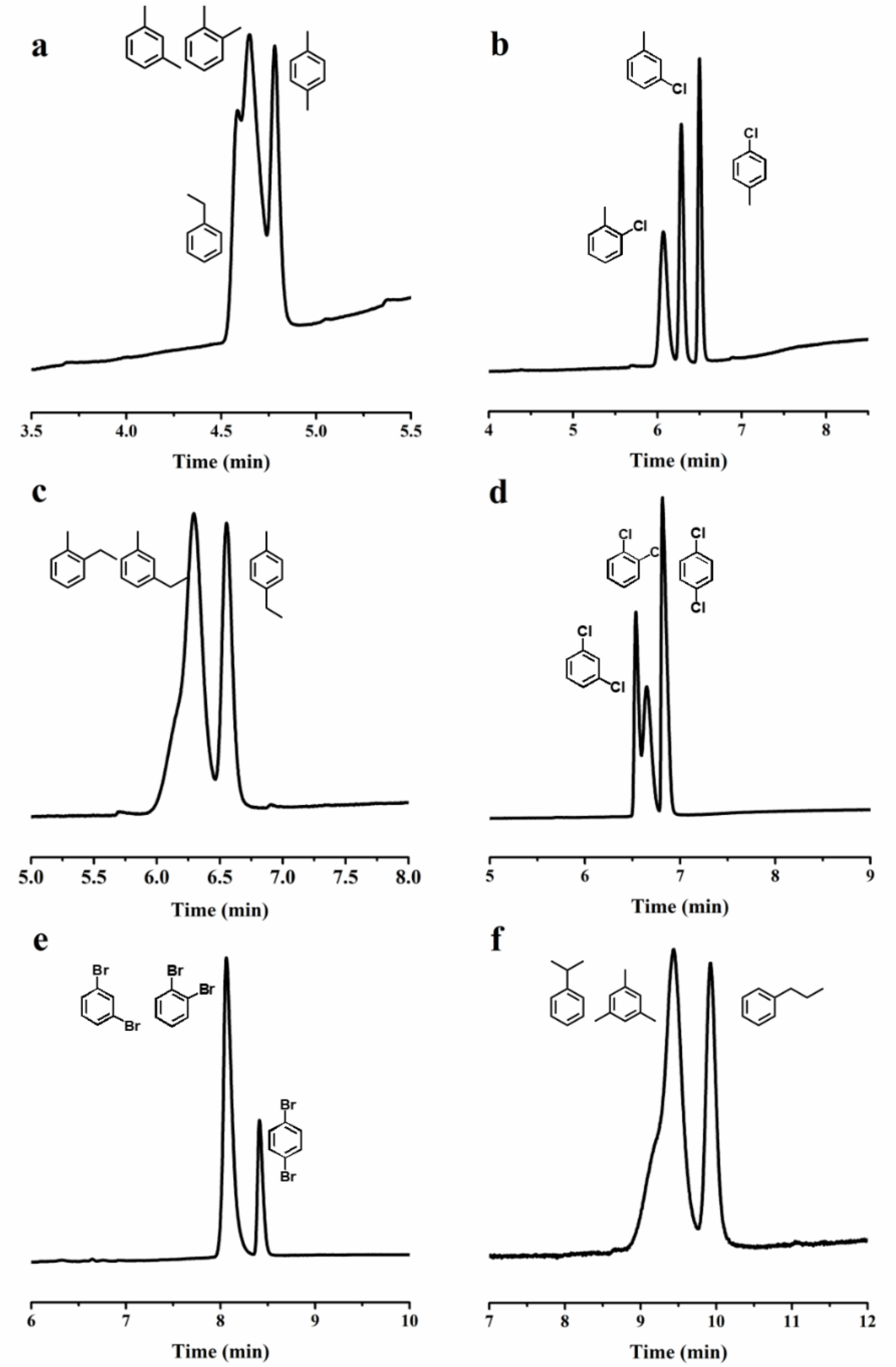

Supplementary Figure 39. Gas chromatograms on the 2-D untwisted Zr-BTB-PABA capillary column (30 m long × 0.25 mm i.d.) under N_2 flow of 1.0 mL·min^{-1} for separation of: (a) xylene isomers and ethylbenzene using a temperature program of 120 ºC for 1 min, and then 30 ºC·min^{-1} to 250 ºC; (b) chlorotoluene isomers using a temperature program of 120 ºC for 1 min, and then 20 ºC·min^{-1} to 250 ºC; (c) ethyltoluene isomers using a temperature program of 120 ºC for 1 min, and then 20 ºC·min^{-1} to 250 ºC; (d) dichlorobenzene isomers using a temperature program of 120 ºC for 1 min, and then 20 ºC·min^{-1} to 250 ºC; (e) dibromobenzene isomers using a temperature program of 120 ºC for 1 min, and then 20 ºC·min^{-1} to 250 ºC; (f) mixtures of n-propylbenzene, isopropylbenzene and 1,3,5-trimethylbenzene using a temperature program of 120 ºC for 1 min, and then 10 ºC·min^{-1} to 250 ºC.

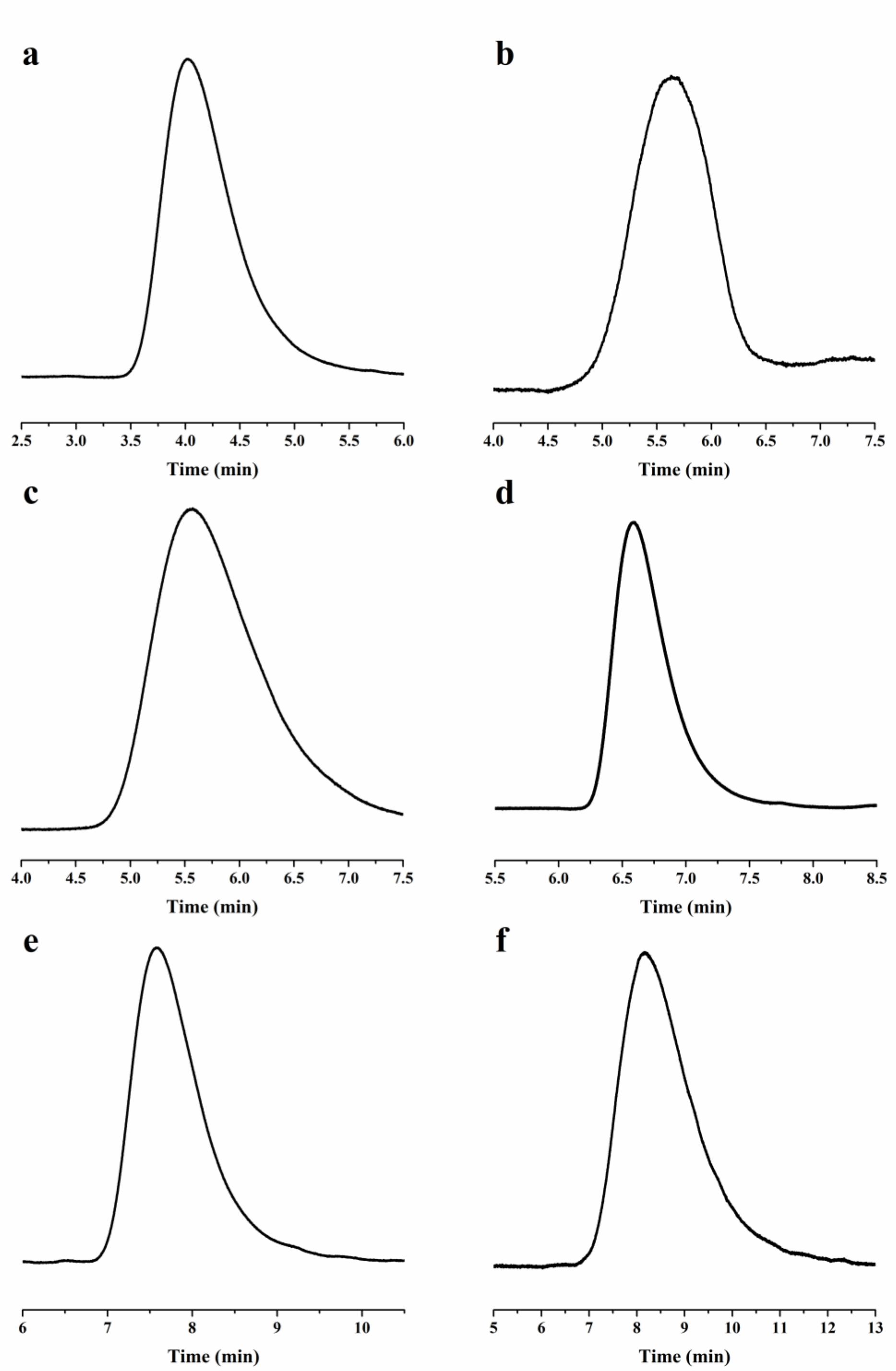

Supplementary Figure 40. Gas chromatograms on the 2-D twisted Zr-BTB-PABA capillary column (30 m long × 0.25 mm i.d.) under N_2 flow of 1.0 mL·min^{-1} for separation of: (a) xylene isomers and ethylbenzene using a temperature program of 100 ºC for 1 min, and then 30 ºC·min^{-1} to 250 ºC; (b) chlorotoluene isomers using a temperature program of 100 ºC for 1 min, and then 20 ºC·min^{-1} to 250 ºC; (c) ethyltoluene isomers using a temperature program of 100 ºC for 1 min, and then 20 ºC·min^{-1} to 250 ºC; (d) dichlorobenzene isomers using a temperature program of 120 ºC for 1 min, and then 20 ºC·min^{-1} to 250 ºC; (e) dibromobenzene isomers using a temperature program of 120 ºC for 1 min, and then 20 ºC·min^{-1} to 250 ºC; (f) mixtures of n-propylbenzene, isopropylbenzene and 1,3,5-trimethylbenzene using a temperature program of 120 ºC for 1 min, and then 10 ºC·min^{-1} to 250 ºC.

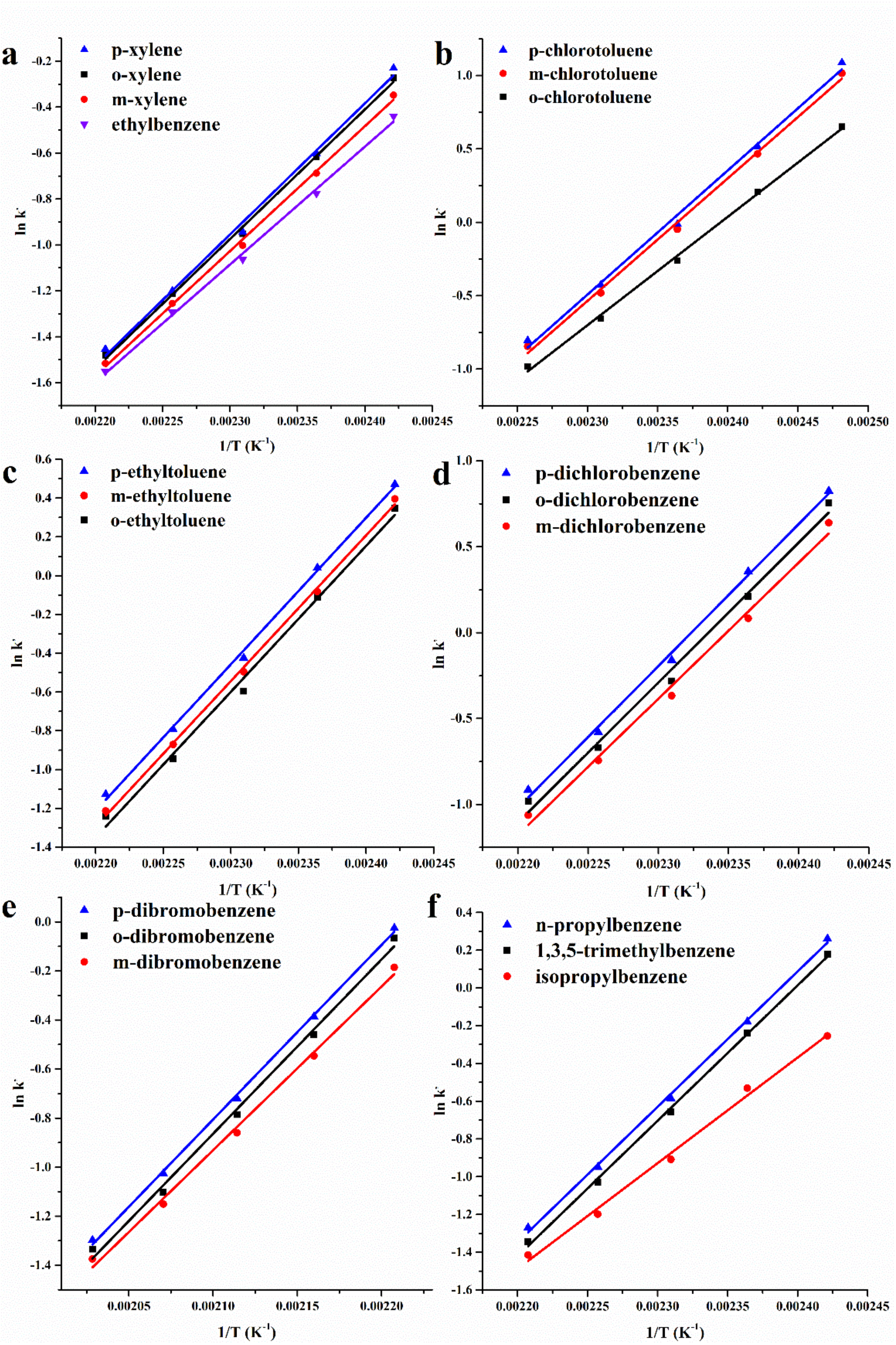

Supplementary Figure 41. van't Hoff plots for the GC separation of structural isomers of benzene derivatives on the 2-D untwisted Zr-BTB-BA nanosheets coated capillary column.

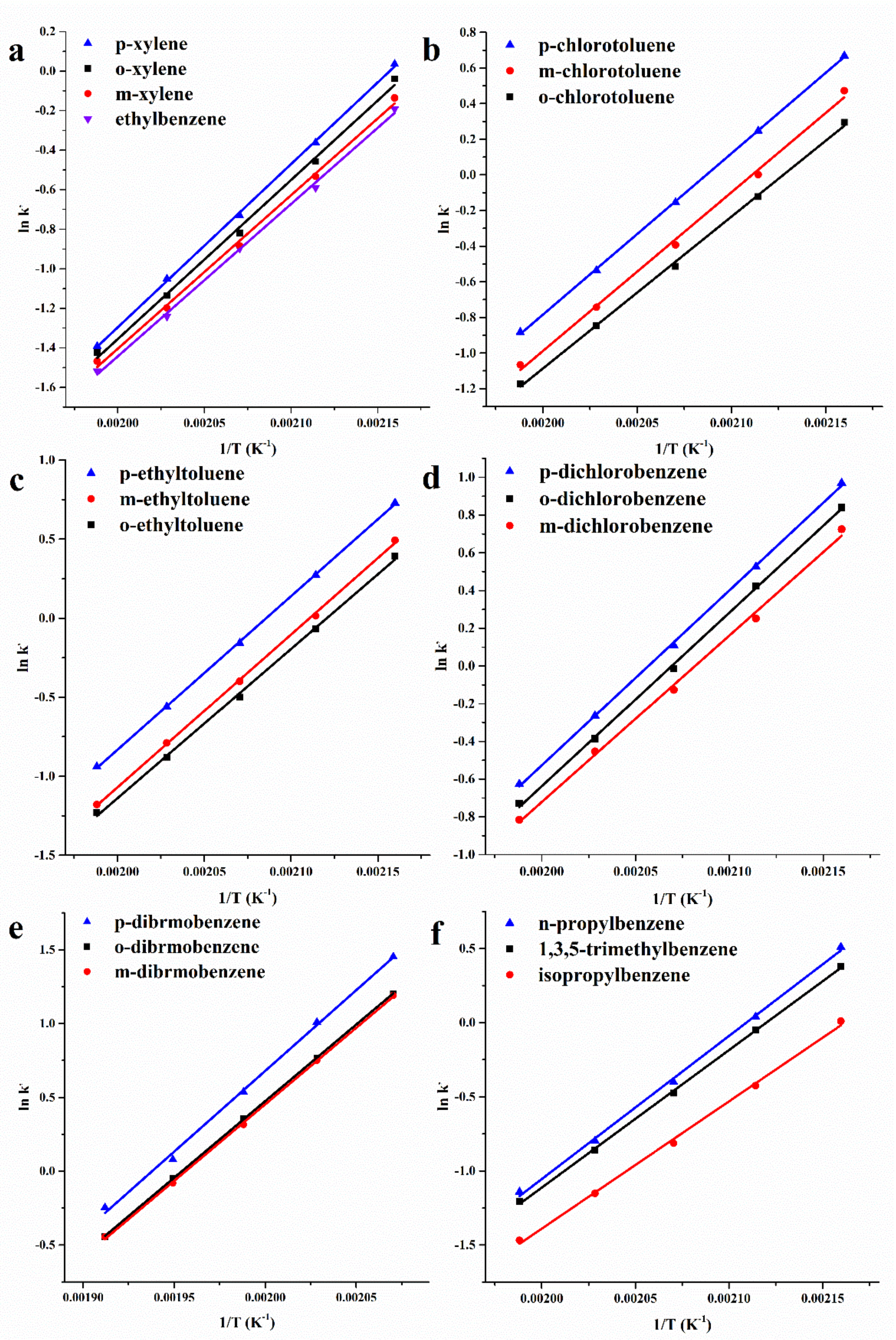

Supplementary Figure 42. van't Hoff plots for the GC separation of structural isomers of benzene derivatives on the 2-D untwisted Zr-BTB-PABA nanosheets coated capillary column.

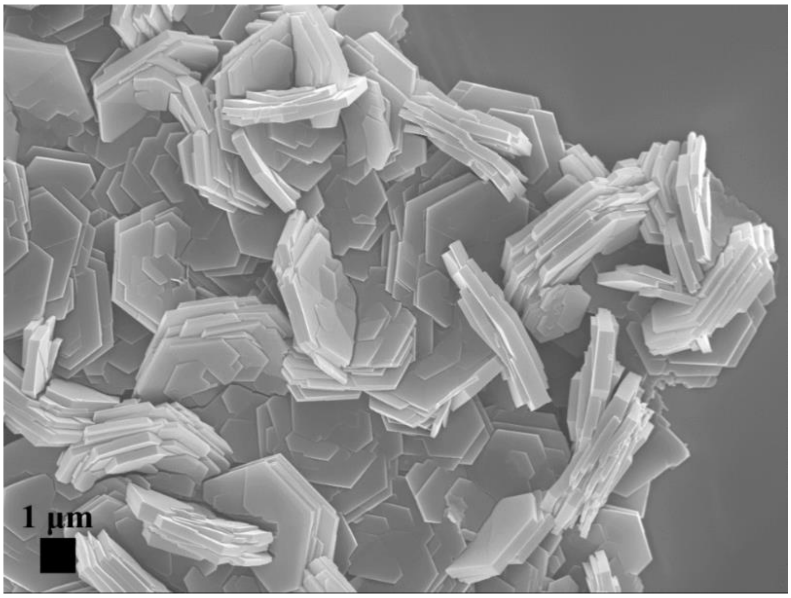

Supplementary Figure 43. SEM image of as-synthesized Zr-BTB micrometer-sized plates.

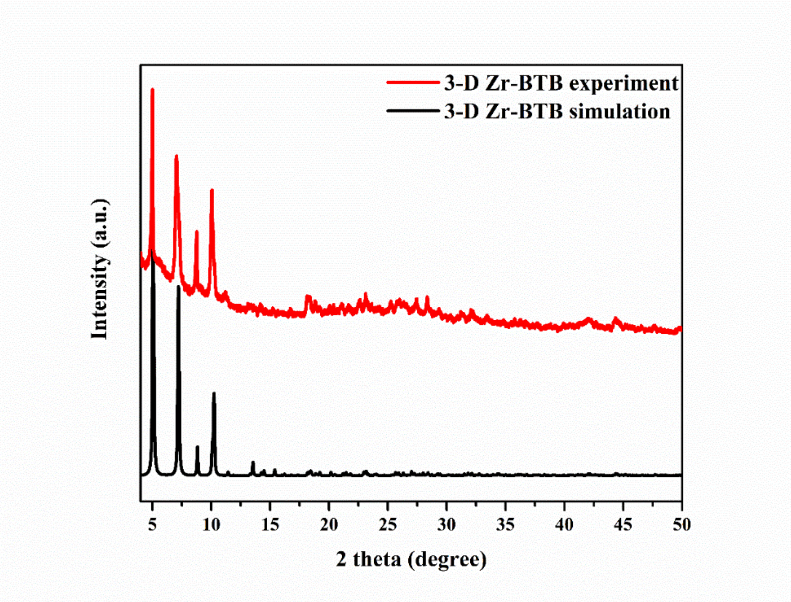

Supplementary Figure 44. The PXRD pattern of as-synthesized 3-D Zr-BTB.

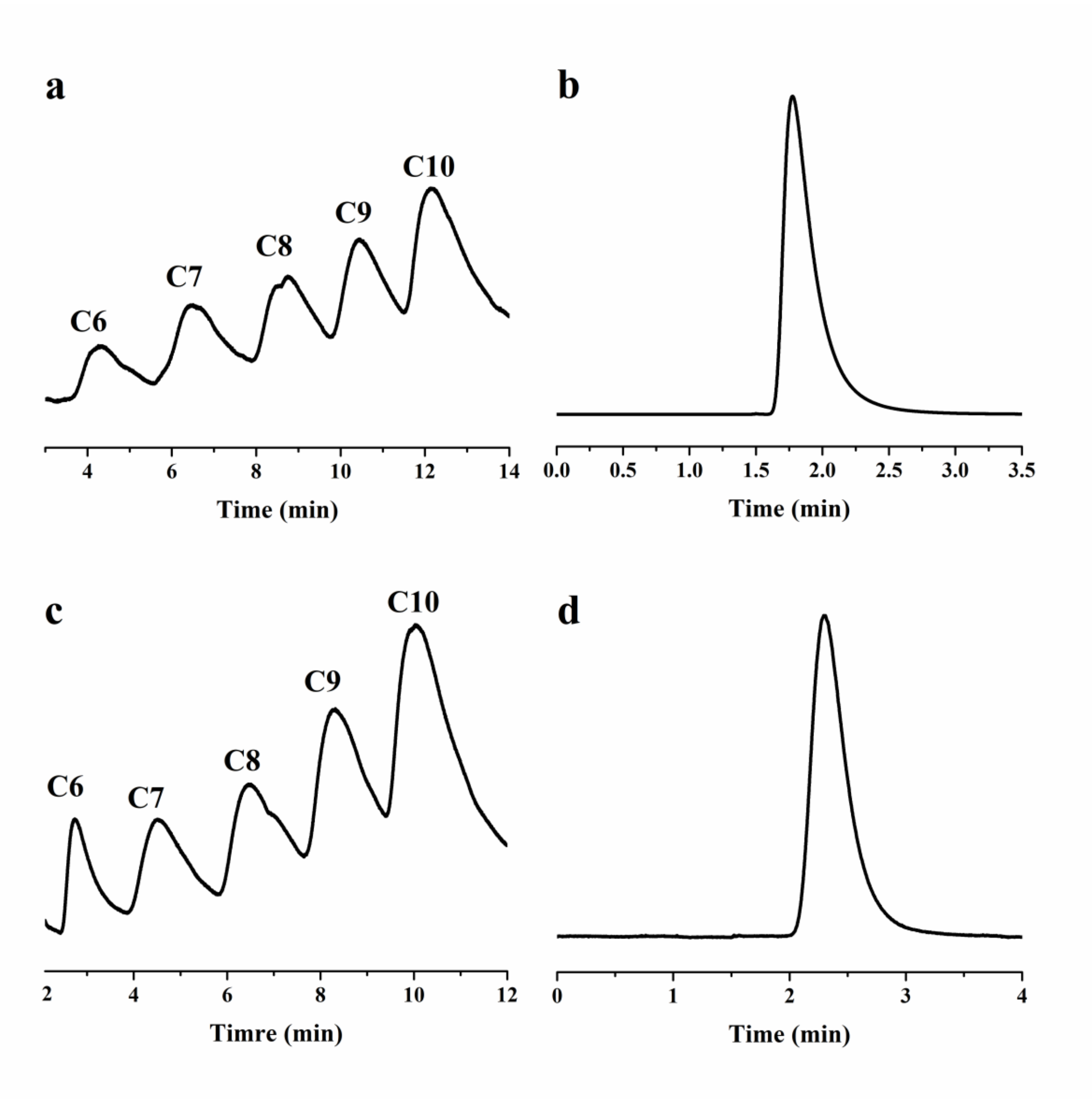

Supplementary Figure 45. Gas chromatograms on the Zr-BTB-FA micrometer-sized plates and 3-D Zr-BTB coated capillary column (30 m long × 0.25 mm i.d.) under N_2 flow of 1.0mL·min^{-1} for separation of (a) linear alkanes using a temperature program of 70 ºC for 1 min, and then 10 ºC·min^{-1} to 250 ºC on the Zr-BTB-FA micrometer-sized plates; (b) xylene isomers and ethylbenzene using a temperature program of 100 ºC for 1 min, and then 20 ºC·min^{-1} to 250 ºC on the Zr-BTB-FA micrometer-sized plates; (c) linear alkanes using a temperature program of 70 ºC for 1 min, and then 10 ºC·min^{-1} to 250 ºC on the 3-D Zr-BTB; (d) xylene isomers and ethylbenzene using a temperature program of 100 ºC for 1 min, and then 20 ºC·min^{-1} to 250 ºC on the 3-D Zr-BTB.

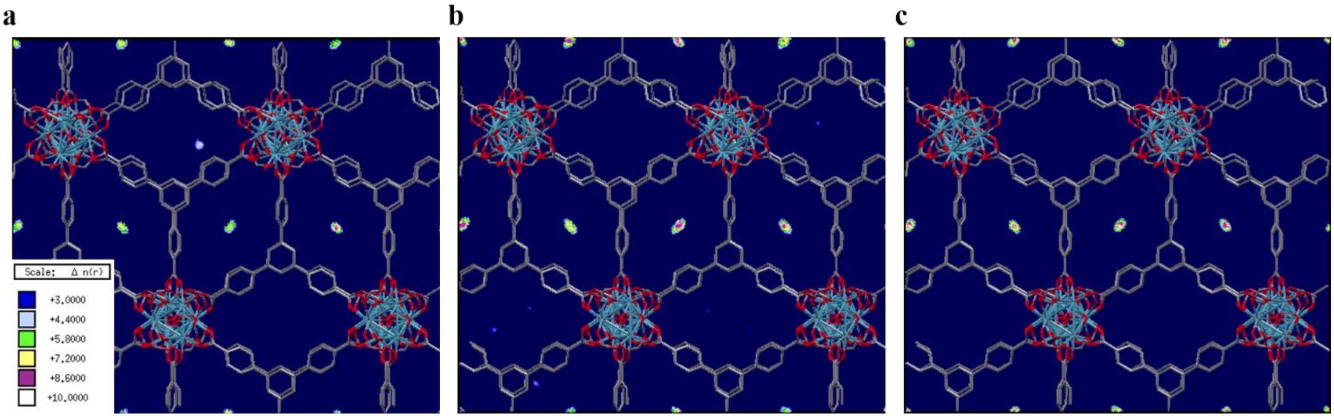

Supplementary Figure 46. Snapshots of density distribution for *p*-xylene adsorbed in the untwisted Zr-BTB-FA nanosheets at 523 K and 10^5 Pa obtained from GCMC simulation: (a) ethylbenzene, (b) m-xylene and (c) o-xylene.

Supplementary Tables

Supplementary Table 1. Columns repeatability and reproducibility on the selectivity and resolution for *meta*- and *para*- isomers on the 2-D untwisted Zr-BTB-FA column, twisted Zr-BTB-FA column, HP-5MS column and VF-WAXms column. (n=7)

	HP-5MS column				VF-WAXMS column				untwisted Zr-BTB-FA column				twisted Zr-BTB-FA column			
	α	RSD(%)	R_s	RSD(%)	α	RSD(%)	R_s	RSD(%)	α	RSD(%)	R_s	RSD(%)	α	RSD(%)	R_s	RSD(%)
xylene	1	0	0	0	1.02	0.06%	0.47	2.74%	1.09	0.09%	4.69	2.73%	1	0	0	0
chlorotoluene	1.02	0.05%	0.62	3.37%	1	0	0	0	1.10	0.01%	3.02	1.06%	1	0	0	0
ethyltoluene	1.01	0.03%	0.28	3.82%	1	0	0	0	1.09	0.08%	2.32	1.90%	1	0	0	0
dichlorobenzene	1.02	0.06%	0.93	2.21%	1.08	0.10%	1.32	2.31%	1.13	0.12%	5.41	1.91%	1	0	0	0
dibromobenzene	1.01	0.02%	0.39	2.98%	1.05	0.07%	0.97	2.06%	1.22	0.05%	6.24	1.29%	1	0	0	0

Supplementary Table 2. Values of ΔH and ΔS for structural isomers of benzene derivatives on the 2-D twisted and untwisted Zr-BTB-FA nanosheets coated capillary column.

	untwisted Zr-BTB-FA coated column		twisted Zr-BTB-FA coated column	
Analytes	ΔH ($kJ\cdot mol^{-1}$)	ΔS ($J\cdot mol^{-1}\cdot K^{-1}$)	ΔH ($kJ\cdot mol^{-1}$)	ΔS ($J\cdot mol^{-1}\cdot K^{-1}$)
o-xylene	-80.47 ± 0.06	-126.94 ± 0.13	-66.80 ± 1.55	-100.17 ± 3.24
m-xylene	-80.41 ± 0.23	-126.97 ± 0.48	-65.43 ± 3.09	-96.92 ± 6.40
p-xylene	-81.45 ± 0.27	-126.25 ± 0.56	-72.22 ± 0.99	-108.48 ± 2.08
ethylbenzene	-79.43 ± 0.28	-125.22 ± 0.58	-65.35 ± 1.63	-97.92 ± 3.41
o- chlorotoluene	-80.07 ± 0.59	-122.22 ± 1.21	-52.24 ± 1.00	-72.32 ± 2.08
m- chlorotoluene	-82.52 ± 0.27	-125.62 ± 0.57	-70.80 ± 0.74	-106.99 ± 1.50
p- chlorotoluene	-83.24 ± 0.88	-124.34 ± 1.83	-72.03 ± 1.22	-105.74 ± 2.50
o- ethyltoluene	-88.50 ± 0.31	-138.69 ± 0.65	-64.97 ± 2.10	-101.41 ± 4.32
m- ethyltoluene	-88.90 ± 0.12	-138.05 ± 0.24	-85.35 ± 2.59	-134.01 ± 5.32
p- ethyltoluene	-89.12 ± 0.03	-135.09 ± 0.06	-85.85 ± 2.18	-130.93 ± 4.49
o- dichlorobenzene	-84.08 ± 0.95	-126.00 ± 1.97	-76.81 ± 1.92	-113.80 ± 3.99
m- dichlorobenzene	-83.50 ± 0.73	-125.78 ± 1.52	-76.19 ± 0.93	-113.05 ± 1.91
p- dichlorobenzene	-84.21 ± 0.47	-123.99 ± 0.97	-80.07 ± 1.53	-118.96 ± 3.16
o- dibromobenzene	-93.14 ± 1.23	-133.14 ± 2.45	-57.18 ± 1.46	-66.75 ± 2.91
m- dibromobenzene	-91.94 ± 0.34	-131.19 ± 0.69	-84.82 ± 3.16	-120.54 ± 6.32
p- dibromobenzene	-93.16 ± 0.05	-130.28 ± 1.26	-90.48 ± 3.55	-127.61 ± 7.07
n-propylbenzene	-87.33 ± 0.83	-134.62 ± 1.71	-80.85 ± 1.90	-124.69 ± 3.74
isopropylbenzene	-90.21 ± 0.27	-142.78 ± 0.55	-12.02 ± 0.09	1.26 ± 0.17
1,3,5-trimethylbenzene	-87.03 ± 0.24	-140.27 ± 0.50	-38.62 ± 0.92	-47.71 ± 1.83

Supplementary Table 3. Columns reproducibility on the selectivity and resolution for *meta*- and *para*- isomers on the 2-D untwisted Zr-BTB-BA column, twisted Zr-BTB-BA column, untwisted Zr-BTB-PABA column and twisted Zr-BTB-PABA column. (n=7)

	untwisted Zr-BTB-BA column				twisted Zr-BTB-BA column				untwisted Zr-BTB-PABA column				twisted Zr-BTB-PABA column			
	α	RSD(%)	R_s	RSD(%)	α	RSD(%)	R_s	RSD(%)	α	RSD(%)	R_s	RSD(%)	α	RSD(%)	R_s	RSD(%)
xylene	1.23	0.08%	1.28	1.41%	1	0	0	0	1.05	0.06%	0.91	1.61%	1	0	0	0
chlorotoluene	1.52	0.25%	1.71	0.85%	1	0	0	0	1.05	0.05%	1.39	1.26%	1	0	0	0
ethyltoluene	1.25	0.09%	2.02	1.04%	1	0	0	0	1.06	0.06%	0.78	1.31%	1	0	0	0
dichlorobenzene	1.25	0.04%	2.02	0.92%	1	0	0	0	1.06	0.05%	1.67	1.35%	1	0	0	0
dibromobenzene	1.06	0.24%	0.26	2.92%	1	0	0	0	1.05	0.07%	1.46	1.57%	1	0	0	0

Supplementary Table 4. Values of ΔH and ΔS for structural isomers of benzene derivatives on the 2-D untwisted Zr-BTB-BA and Zr-BTB-PABA nanosheets coated capillary column.

	untwisted Zr-BTB-BA coated column		untwisted Zr-BTB-PABA coated column	
Analytes	ΔH ($kJ \cdot mol^{-1}$)	ΔS ($J \cdot mol^{-1} \cdot K^{-1}$)	ΔH ($kJ \cdot mol^{-1}$)	ΔS ($J \cdot mol^{-1} \cdot K^{-1}$)
o-xylene	-46.95 ± 1.11	-68.33 ± 2.56	-66.86 ± 1.97	-97.26 ± 4.08
m-xylene	-45.23 ± 1.05	-64.83± 2.43	-64.58 ± 1.84	-93.10 ± 3.82
p-xylene	-47.51 ± 1.78	-69.46 ± 4.12	-68.65 ± 0.86	-100.34 ± 1.75
ethylbenzene	-42.69 ± 1.21	-59.46 ± 2.80	-64.07 ± 1.44	-92.43 ± 2.99
o- chlorotoluene	-61.39 ± 1.55	-99.29 ± 3.67	-70.89 ± 1.31	-103.08 ± 2.75
m- chlorotoluene	-69.41 ± 2.17	-116.36 ± 5.13	-74.08 ± 2.24	-108.65 ± 4.66
p- chlorotoluene	-70.32 ± 2.56	-118.09 ± 5.99	-75.27 ± 0.59	-109.32 ± 1.25
o- ethyltoluene	-62.53 ± 2.72	-101.05 ± 6.30	-78.55 ± 1.51	-118.79 ± 3.16
m- ethyltoluene	-62.44 ± 1.38	-100.39 ± 3.19	-80.42 ± 1.01	-121.95 ± 2.08
p- ethyltoluene	-62.82± 1.49	-100.53 ± 3.44	-80.85 ± 0.56	-120.87 ± 1.17
o- dichlorobenzene	-67.81 ± 3.26	-110.64 ± 7.53	-76.53 ± 1.20	-110.65 ± 2.49
m- dichlorobenzene	-65.96 ± 3.14	-107.17 ± 7.26	-73.43 ± 2.10	-105.08 ± 4.33
p- dichlorobenzene	-68.77 ± 2.14	-112.06 ± 4.96	-77.15 ± 0.90	-110.98± 1.92
o- dibromobenzene	-59.11 ± 2.20	-83.58 ± 4.66	-86.30 ± 0.42	-120.95 ± 0.83
m- dibromobenzene	-55.50 ± 1.92	76.56 ± 4.07	-86.20 ± 0.87	-120.87 ± 1.75
p- dibromobenzene	-59.21 ± 1.02	-83.29 ± 2.16	-90.95 ± 2.42	-128.52 ± 4.82
n-propylbenzene	-59.69 ± 1.21	-94.78 ± 2.80	-80.27 ± 1.70	-121.54 ± 3.50
Isopropylbenzene	-46.54 ± 1.91	-67.03 ± 4.42	-71.39 ± 1.73	-106.57 ± 3.58
1,3,5-trimethylbenzene	-59.67 ± 1.10	-95.36 ± 2.55	-77.13 ± 0.94	-115.72 ± 1.92

Supplementary Table 5. Simulated adsorption capacities of xylenes isomers and ethylbenzene in 2-D untwisted Zr-BTB-FA nanosheets.

	ethylbenzene	m-xylene	o-xylene	p-xylene
Molecules/UC	15	17	17	18

Supplementary Table 6. Simulated adsorption capacities of xylenes isomers and ethylbenzene in 2-D twisted Zr-BTB-FA nanosheets.

	ethylbenzene	m-xylene	o-xylene	p-xylene
Molecules/UC	32	32	32	32

Supplementary References

1. Hu Z, Mahdi EM, Peng Y, Qian Y, Zhang B, Yan N*, et al.* Kinetically controlled synthesis of two-dimensional Zr/Hf metal–organic framework nanosheets via a modulated hydrothermal approach. *J. Mater. Chem. A* **5,** 8954-8963 (2017).

2. Yang D, Bernales V, Islamoglu T, Farha OK, Hupp JT, Cramer CJ*, et al.* Tuning the surface chemistry of metal organic framework nodes: proton topology of the metal-oxide-like Zr6 nodes of UiO-66 and NU-1000. *J. Am. Chem. Soc.* **138,** 15189-15196 (2016).

3. Yang D, Ortuño MA, Bernales V, Cramer CJ, Gagliardi L, Gates BC. Structure and dynamics of Zr_6O_8 metal–organic framework node surfaces probed with ethanol dehydration as a catalytic test reaction. *J. Am. Chem. Soc.* **140,** 3751-3759 (2018).
